# Cytotoxic 13,28 Epoxy Bridged Oleanane-Type Triterpenoid Saponins from the Roots of *Ardisia crispa*

**DOI:** 10.3390/molecules27031061

**Published:** 2022-02-04

**Authors:** Xin Yin, Ruihang Hu, Yongqiang Zhou, Weiqian Zhu, Ying Zhou

**Affiliations:** College of Pharmacy, Guizhou University of Traditional Chinese Medicine, Guiyang 550025, China; yinxin110901@163.com (X.Y.); huruihang1997@163.com (R.H.); zhouxiaoqiang1988@126.com (Y.Z.); zhuwq0820@163.com (W.Z.)

**Keywords:** *Ardisia*, oleanane-type triterpenoid, cytotoxicity, natural product

## Abstract

Ardisiacrispin D–F (**1**–**3**), three new 13,28 epoxy bridged oleanane-type triterpenoid saponins, together with four known analogues (**4**–**7**) were isolated from the roots of *Ardisia crispa*. The structures of **1**–**7** were elucidated based on 1D and 2D-NMR experiments and by comparing their spectroscopic data with values from the published literatures. Ardisiacrispin D–F (**1**–**3**) are first examples that the monosaccharide directly linked to aglycone C-3 of triterpenoid saponins in genus *Ardisia* are non-arabinopyranose. In the present paper, all compounds are evaluated for the cytotoxicity against three cancer cell lines (HeLa, HepG2 and U87 MG) in vitro. The results show that compounds **1**, **4** and **6** exhibited significant cytotoxicity against Hela and U87 MG cells with IC_50_ values in the range of 2.2 ± 0.6 to 9.5 ± 1.8 µM. The present investigation suggests that roots of *A. crispa* could be a potential source of natural anti-tumor agents and their triterpenoid saponins might be responsible for cytotoxicity.

## 1. Introduction

The genus *Ardisia* (Primulaceae) is widely distributed in subtropical and tropical regions of the world, and, for along time, its roots have primarily been used as traditional medicines [1]. In previous phytochemical investigations, the chemical constituents of the genus *Ardisia* were reported to be saponins, isocoumarins, peptides, quinones and alkylphenols [1]. The roots of *Ardisia* species appeared to be a rich source of triterpenoid saponins, which have been isolated from various *Ardisia* species, including *A. crenata*, *A. crispa*, *A. mamillata* and *A. pusilla*. For example, novel triterpenoid saponins, ardisicrenosides A-B and ardisiacrispins A-B, were previously isolated from *A. crenata* and *A. crispa*, respectively [1]. Phytochemical investigations revealed that triterpenoid saponins are the main constituents of the genus *Ardisia*, and have significant cytotoxic activities [2,3,4,5,6,7,8]. Triterpenoid saponins reported from *Ardisia* plants are interesting from the viewpoints of chemical diversity and biological activity.

The roots of *Ardisia crispa* are utilized as traditional Chinese medicine for treating a sore throat, damp-heat jaundice and bruises [9]. Previous pharmacological studies on this plant have revealed that it has anti-tumor, anti-inflammatory and suppressed angiogenesis effects [10,11,12,13,14,15,16,17,18], but its chemical composition is rarely reported. In order to find more potentially cytotoxic triterpenoid saponins from *A. crispa*, an extract of the roots of this species was chemically investigated. Herein, the isolation, structural elucidation and the cytotoxic activities of the triterpenoid saponins are discussed.

## 2. Results and Discussion

**Figure 1:**
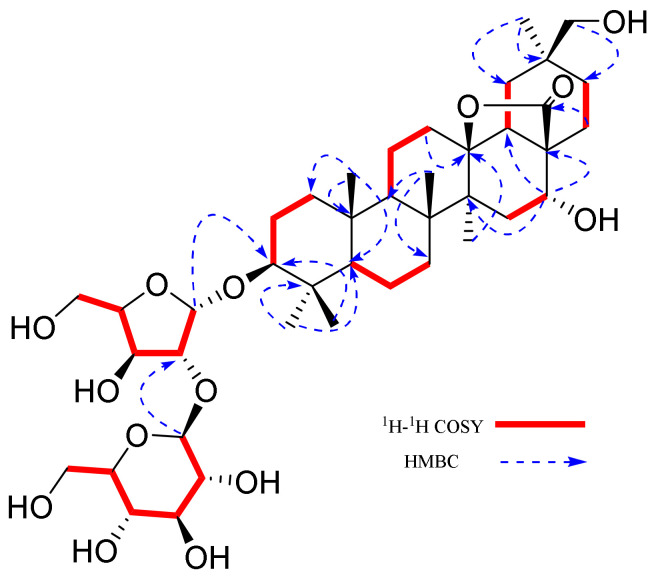
Key HMBC and ^1^H-^1^H COSY correlations of ardisiacrispin D (**1**).

Compound **1** was obtained as a white amorphous powder. Its molecular formula was deduced as C_41_H_66_O_14_ by HR-ESI-MS at *m/z* 805.4317 [M + Na]^+^ (calcd. for C_41_H_66_O_14_Na, 805.4344). The ^1^H-NMR spectrum of **1** (Table 1) reveals the presence of 6 methyl proton signals of a typical oleanane-type triterpenoid skeleton [19] at *δ*_H_ 0.99 (3H, s, H_3_-23), 0.78 (3H, s, H_3_-24), 0.91 (3H, s, H_3_-25), 1.08 (3H, s, H_3_-26), 1.40 (3H, s, H_3_-27), 0.92 (3H, s, H_3_-29); and 2 oxygen-bearing methylene protons *δ*_H_ 3.55 (1H, d, *J* = 11.1 Hz, H-30a), 3.25 (1H, d, *J* = 11.1 Hz, H-30b), together with 2 anomeric proton signals *δ*_H_ 5.23 (1H, brs, H-1′) and *δ*_H_ 4.44 (1H, d, *J* = 7.8 Hz, H-1″). The above ^1^H-NMR data, together with a carbonyl carbon signal at (*δ*_C_ 181.0, C-28) and an oxygenated quaternary carbon at (*δ*_C_ 95.0, C-13) in the ^13^C-NMR spectrum, suggested **1** to be a 13,28 epoxy bridged oleanane-type triterpenoid skeleton in the aglycone and the presence of 2 sugar units [19]. The ^13^C-NMR data of the aglycon in **1** was similar to ardisicrenoside A, which was obtained previously from the *Ardisia crenata*, except for the presence of a carbon signal of *γ*-lactone moiety at C-28 (*δ*_C_ 181.0) in **1**, rather than a oxygenated methylene carbon signal (*δ*_C_ 77.5) in ardisicrenoside A [19]. The identity of the monosaccharides and the linkage of the sugar residues were made by the combination of DEPT-135, HSQC, ^1^H-^1^H COSY and HMBC spectra. The connectivity of the 2 sugars was mainly based on the HMBC correlations: H-1′ (*δ* 5.23, 1H, brs) with C-3 (*δ*_C_ 89.4) of the aglycone, H-1″ (*δ*_H_ 4.44, 1H, d, *J* = 7.8 Hz) with C-2′ (*δ*_C_ 89.6) (Figure 1). By comparing the chemical shift signals and coupling constants of the sugar moieties from the published literature [20], the relative configurations of the anomeric centers of the arabinosyl and glucosyl moieties in **1** were determined to be *α* and *β*, respectively. The two kinds of sugars, l-arabinofuranose and d-glucose, were identified by GC analysis after derivatization.

**Figure 2:**
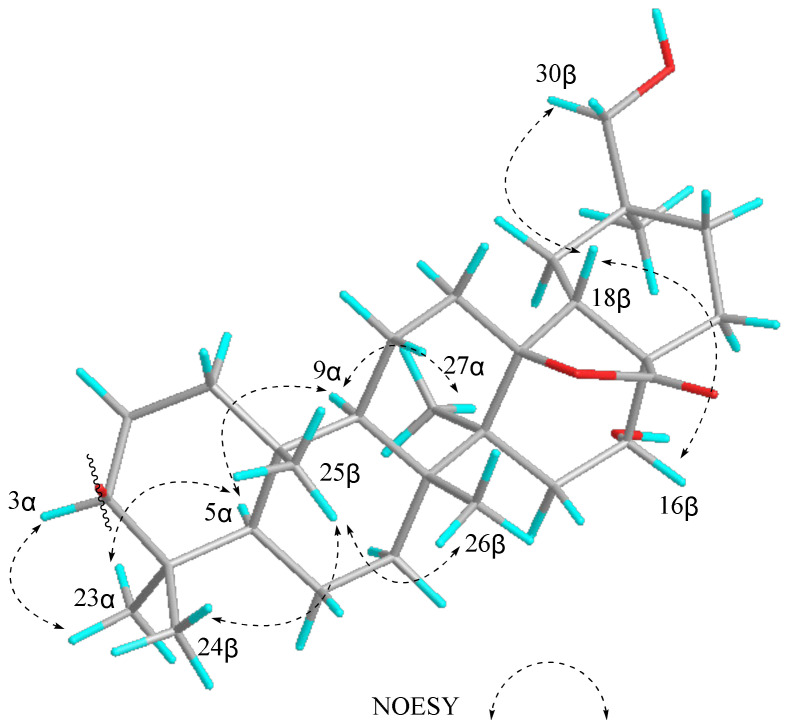
Key NOESY correlations of the aglycon of ardisiacrispin D (**1**).

**Figure 3:**
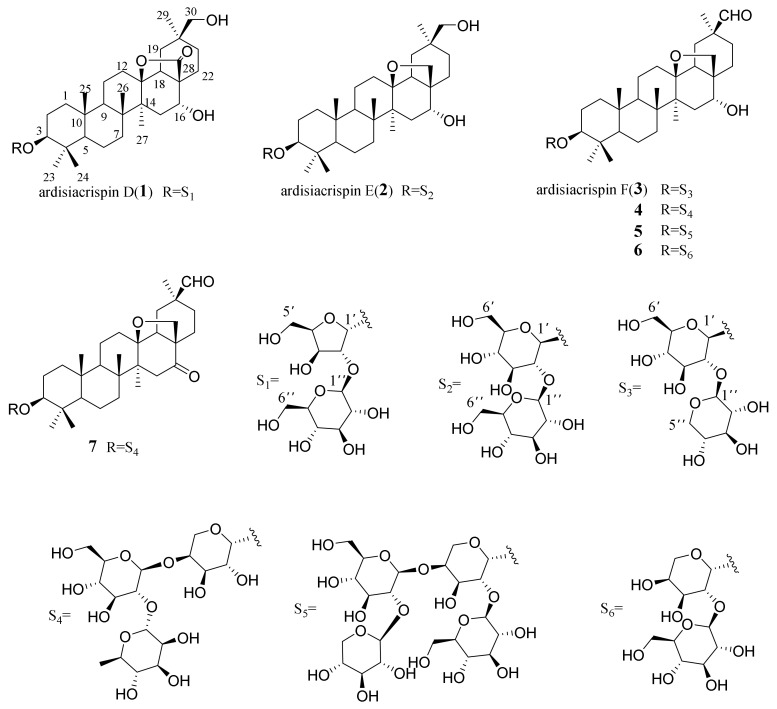
Chemical structures of compounds **1**–**7.**

The NOESY correlations (Figure 2) of Me-23α/H-3*α*, H-5*α*; H-5*α*/H-9*α*; H-9*α*/Me-27*α*; Me-24*β*/Me-25*β*, Me-25*β*/Me-26*β*; H-18*β*/H-16*β*, H-30*β* indicated the *α*-orientation of H-3 and *β*-orientation of H-16. Thus, **1** was inferred as 3*β*, 16*α*, 30-trihydroxyolean-13*β*,28-olide-3-O-*β*-D-glucopyranosyl-(1→2)-*α*-l-arabinofuranose, and named as ardisiacrispin D (Figure 3).

Compound **2** was separated as a white amorphous powder. The molecular formula of **2** was established to be C_42_H_70_O_14_ from its HR-ESI-MS at *m/z* 821.4628 [M + Na]^+^ (calcd. for 821.4657). In the ^1^H and ^13^C-NMR spectrum of **2** (Table 1), signals due to an aglycone moiety were similar with those of ardisicrenoside B [19], although the signals due to the sugar moiety were not identical. The NMR data of **2** showed two anomeric proton signals at *δ*_H_ 4.39 (1H, d, *J* = 7.8 Hz, H-1′) and 4.65 (1H, d, *J* = 7.7 Hz, H-1″), corresponding to two anomeric carbons at *δ*_C_ 105.9 and 104.6 in the HSQC spectrum, indicating the presence of two sugar moieties. The attachments of the sugar chain were deduced from the HMBC spectrum. The HMBC showed that there was a correlation between H-1′ (*δ*_H_ 4.39, 1H, d, *J* = 7.8 Hz) and C-3 (*δ*_C_ 91.3) of the aglycone; H-1″ (*δ*_H_ 4.65, 1H, d, *J* = 7.7 Hz) and C-2′ (*δ*_C_ 79.1) indicated that the sugar chain was connected to C-3 of the aglycone, and the terminal sugar was linked to C-2′. The anomeric configuration of these 2 glucoses were determined to be *β* on the basis of the *J* value of the anomeric proton in glucose (*J* = 7.8 and 7.7 Hz)]. The relative configurations of **2** were determined by the NOESY experiment, which were consistent with those of **1**. On the basis of above evidence, the structure of **2** was identified to be 3*β*, 16*α*, 30-trihydroxyolean-13*β*,28-epoxy-3-*O*-*β*-d-glucopyranosyl-(1→2)-*β*-d-glucopyranose, and named as ardisiacrispin E.

Compound **3** was isolated as white amorphous powder. The molecular formula was inferred as C_41_H_66_O_13_ according to the positive-ion HR-ESI-MS peak at *m/z* 789.4367 [M + Na]^+^ (calcd for C_41_H_66_O_13_Na, 789.4395). The NMR spectra of the aglycone of **3** closely resembled that of **2** (Table 1), except for ^13^C-NMR signals of C-20 (*δ*_C_ 36.9), C-29 (*δ*_C_ 28.5) and C-30 (*δ*_C_ 66.5) in **2**, which were shifted to *δ*_C_ 49.2, 24.3 and 209.2 in **3**, respectively. Moreover, the singlet observed at *δ*_H_ 9.40 was assigned as a formyl proton. The above NMR data suggested that C-30 in **3** was substituted with a formyl group, which was supported by the HMBC correlations from H-29/H-30 to C-20 and from H-29 to C-19, C-21 and C-30. The NMR data for the sugar moiety and GC analysis of the derivatives of the hydrolyzate of **3** indicated the existence of d-xylopyranose and d-glucopyranose. The anomeric proton signals of **3** at *δ*_H_ 4.40 (1H, d, *J* = 7.0 Hz, H-1′) and 4.65 (1H, d, *J* = 7.7 Hz, H-1″), led to the assignment of the anomeric configurations of glucose and xylose units as *β* due to their relatively large coupling constants (*J* values > 7). In the HMBC spectrum, the long-range correlations of *δ*_H_ 4.40 (1H, d, *J* = 7.0 Hz, H-1′) with *δ*_C_ 91.3 (C-3) and *δ*_H_ 4.65(1H, d, *J* = 7.7 Hz, H-1″) with *δ*_C_ 80.9 (C-2′) were observed. The ^1^H and ^13^C-NMR spectra of the sugar parts of **3** were in accordance with those of majonoside-R2 [21]. The relative configurations of **3** were determined by the NOESY experiment, which were consistent with those of **2**. Therefore, the structure of **3** was identified as 3*β*, 16*α* dihydroxyolean-13*β*,28-epoxy-30-al-3-*O*-*β*-d-xylopyranosyl-(1→2)-*β*-d-glucopyranose, and named as ardisiacrispin F.

By NMR data analysis and comparison with the reported spectroscopic data, four known compounds were identified as 3-*O*-*α*-l-rhamnopyranosyl-(1→2)-*β*-d-glucopyranosyl-(1→4)-*α*-l-arabinopyranosyl-cyclamiretin A (**4**) [22]; ardisiacrispin A (**5**) [19]; 3-*O*-*β*-d-glucopyranosyl-(1→2)-*α*-l-arabinopyranoside-cyclamiretin A (**6**) [23]; and ardisimamillosides H (**7**) [24] (Supporting Information).

To date, the monosaccharide directly linked to aglycone C-3 of triterpenoid saponins reported in *Ardisia* are all arabinopyranose. In this study, we reported three novel triterpenoid saponins from *Ardisia crispa* with arabinofuranosyl and glucosyl groups directly connected to aglycone C-3 for the first time. Therefore, the above results are of great significance to elucidate the biosynthesis of triterpenoid saponins in genus *Ardisia*.

In addition, all the isolates (**1**–**7**) were tested for their cytotoxicity against the HeLa, HepG2 and U87 MG cell lines using the MTT method. The results (Table 2) show that the compounds (**1**, **4** and **6**) exhibited more significant cytotoxic activities than cisplatin (Sigma, >99%), which was used as a positive control. Due to the limited numbers of active compounds, only the superficial and primary structure–activity relationships were discussed. Compared with compounds **4**–**7**, **1**–**3** showed stronger cytotoxicity against Hela cells, which suggested that the non-arabinopyranose directly linked to aglycone C-3 might make a contribution to their cytotoxicity. However, The IC_50_ values of compounds **2** and **3** on HepG2 cells were higher than **4**–**6**, which might be caused by the arabinopyranosyl group in **4**–**6**. Compound **5** exhibited better cytotoxic activity against U87 MG cells compared with **1**–**4** and **6**–**7**, which demonstrated that the number of monosaccharide may affect the activity.

## 3. Materials and Methods

### 3.1. General Experimental Procedures

Optical rotations were measured on a JASCO P-2000 instrument. The 1D and 2D NMR spectra were recorded on either a Bruker DPX 400 instrument with tetramethylsilane as an internal standard and MeOH-*d*_4_ as a solvent. HR-ESI-MS experiments were conducted using a Thermo Fisher QE Focus spectrometer. The semi-preparative HPLC procedure was conducted on a Shimadzu LC-16D instrument with an RID-20A (reflective index detector) and a reversed-phase C_18_ column (250 × 10 mm, 5 μm, Waters SunFire). Column chromatography was performed using silica gel (200–300 mesh, China), octadecyl silica (ODS) (50 μm, Merck, Germany).

### 3.2. Plant Material

The roots of *Ardisia crispa* were collected from Kaili of Guizhou Province (China), and identified by Professor Sheng-Hua Wei from Guizhou University of Traditional Chinese Medicine. The voucher specimen (No. 20190502) was deposited at Guizhou University of Traditional Chinese Medicine.

### 3.3. Extraction and Isolation

The air-dried *A. crispa* roots (7.0 kg) were extracted under reflux with 70% EtOH. The filtrates were concentrated on a rotary evaporator (EYELA, N-1300 type) under reduced pressure to yield 1900 g of crude extract. The crude extract was partitioned with petroleum ether, EtOAc and *n*-BuOH successively, yielding *n*-BuOH (360.0 g) extracts. The *n*-BuOH-rich fraction (290 g) was subjected to silica gel column chromatography eluting with gradient mixtures of CH_2_Cl_2_-CH_3_OH (1:0-0:1) to produce compound **5** (56.3 g) and 9 main fractions (Fr. A−Fr. I). Fraction G (12.0 g) was chromatographed on an ODS column with MeOH-H_2_O (1:9 to 1:0) to afford sub-fractions G1–G7. Sub-fraction G5 (870 mg) was separated by semi-preparative HPLC (MeOH-H_2_O, 71:29; flow rate: 3 mL·min^−1^) to create compound **7** (9.2 mg, *t_R_* 16.5 min), compound **1** (3.4 mg, *t_R_* 18.8 min) and compound **3** (4.2 mg, *t_R_* 21.5 min). Sub-fraction G6 (620 mg) was purified by semi-preparative HPLC (MeOH-H_2_O, 78:22; flow rate: 3 mL·min^−1^) to yield compound **4** (11.0 mg, *t_R_* 15.4 min), compound **2** (3.8 mg, *t_R_* 18.7 min) and compound **6** (13.0 mg, *t_R_* 21.6 min).

### 3.4. Characterization of Compounds ***1***–***3***

Ardisiacrispin D (**1**): white amorphous powder; ${\left[\alpha \right]}_{D}^{27}$ = −26, (*c* = 0.1, MeOH); HR-ESI-MS *m/z* 805.4317 [M + Na]^+^ (calcd for C_41_H_66_O_14_Na, *m/z* 805.4344); ^1^H-NMR (methanol-*d*_4_, 400 MHz) and ^13^C-NMR (methanol-*d*_4_, 100 MHz), see Table 1.

Ardisiacrispin E (**2**): white amorphous powder; ${\left[\alpha \right]}_{D}^{27}$ = −8, (*c* = 0.05, MeOH); HR-ESI-MS *m/z* 821.4628 [M + Na]^+^ (calcd for C_42_H_70_O_14_Na, *m/z* 821.4657); ^1^H-NMR (methanol-*d*_4_, 400 MHz) and ^13^C-NMR (methanol-*d*_4_, 100 MHz), see Table 1.

Ardisiacrispin F (**3**): white amorphous powder; ${\left[\alpha \right]}_{D}^{27}$ = +4, (*c* = 0.05, MeOH); HR-ESI-MS *m/z* 789.4367 [M + Na]^+^ (calcd for C_41_H_66_O_13_Na, *m/z* 789.4395); ^1^ H-NMR (methanol-*d*_4_, 400 MHz) and ^13^C-NMR (methanol-*d*_4_, 100 MHz), see Table 1.

### 3.5. Acid Hydrolysis of Ardisiacrispin D-F (***1***–***3***)

In order to determine the absolute configuration of the monosaccharide in triterpenoid saponins, the acid hydrolysis of new compounds were performed. The acid hydrolysis of ardisiacrispin D-F (**1**–**3**) (1.0 mg each) was performed according to the previous literatures [20,25,26]. The trimethylsilylthiazolidine derivatives from the *n*-hexane layer were analyzed with GC-MS with a DB-5 capillary column. The absolute configurations of the sugar moieties were established by comparison with the retention times of the authentic sugars (d-xylose, 15.56 min; l-arabinofuranose, 16.40 min; d-glucose, 18.21 min).

### 3.6. Cytotoxic Activity

The cytotoxic activity of compounds (**1**–**7**) against three human cancer cell lines, namely Hela (human cervical cancer cells), HepG-2 (human hepatoma cells) and U87 MG (human glioblastoma cells), were evaluated by the MTT colorimetric assay described in a previous paper [27]. All the cells were cultured in DMEM supplemented with 10% FBS and antibiotics in a humidified atmosphere containing 5% CO_2_ at 37 °C. Briefly, 100 µL of adherent cells were seeded into each well of 96-well cell culture plates at optimal cell density (1 × 10^5^ cells per well) and allowed to adhere for 12 h. The IC_50_ value of each compound was tested on the basis of cell viability after 48 h of treatment with different concentrations of compounds, with cisplatin (Sigma, >99%) as the positive control.

## 4. Conclusions

To summarize, a total of seven 13,28 epoxy bridged oleanane-type triterpenoid saponins were isolated from the roots of *A. crispa* and 3 of them were new structures. In previous investigations, *Ardisia* species appeared to be a rich source of triterpenoid saponins and have significant cytotoxic activities. In this paper, compounds (**1–7**) were evaluated for the cytotoxicity against three cancer cell lines (HeLa, HepG2 and U87 MG) in vitro. Compounds **1, 4** and **6** exhibited significant cytotoxicity against Hela and U87 MG cells with IC_50_ values in the range of 2.2 ± 0.6 to 9.5 ± 1.8 µM. The present investigation suggested that the roots of *A. crispa* could be a potential source of natural anti-tumor agents. Their triterpenoid saponins might be responsible for cytotoxicity, and also seem to be of great chemotaxonomic value for *A. crispa*.

## Figures and Tables

**Table 1:** ^1^H and ^13^C-NMR data of compounds **1–3** in methanol-*d*_4_ (*δ* in ppm).

	1		2		3	
Position	*δ*_C_	*δ*_H_ (*J* in Hz)	*δ*_C_	*δ*_H_ (*J* in Hz)	*δ*_C_	*δ*_H_ (*J* in Hz)
1	40.0	1.78 (m) 0.98 (m)	40.2	1.73 (m) 1.00 (m)	40.2	1.73 (m) 0.97 (m)
2	26.7	1.96 (m) 1.67 (m)	27.3	1.95 (m) 1.70 (m)	27.3	1.85 (m) 1.70 (m)
3	89.4	3.15 (overlap)	91.3	3.16 (overlap)	91.3	3.14 (dd, 11.3, 4.2)
4	40.0	-	40.5	-	40.6	-
5	56.5	0.79 (brd, 7.1)	56.8	0.72 (brd, 11.1)	56.8	0.72 (brd, 9.7)
6	18.8	1.51 (m) 1.46 (m)	18.7	1.51 (m) 1.42 (m)	18.7	1.49 (m) 1.42 (m)
7	34.8	1.59 (m) 1.24 (m)	35.1	1.56 (m) 1.20 (m)	35.1	1.54 (m) 1.20 (m)
8	43.1	-	43.3	-	43.4	-
9	50.8	1.35 (overlap)	51.6	1.46 (overlap)	51.3	1.24 (overlap)
10	37.8	-	37.7	-	37.8	-
11	19.4	1.65 (m) 1.50 (m)	19.8	1.64 (m) 1.48 (m)	19.8	1.63 (m) 1.48 (m)
12	32.1	2.16 (m) 1.56 (m)	32.8	2.05 (m) 1.33 (m)	33.2	2.10 (m) 1.26 (m)
13	95.0	-	88.5	-	88.2	-
14	43.0	-	45.3	-	45.3	-
15	37.4	1.78 (overlap) 1.44 (overlap)	36.9	2.08 (overlap) 1.20 (overlap)	37.0	2.07 (m) 1.20 (m)
16	73.3	3.96 (brd, 5.2)	78.0	3.89 (overlap)	77.8	3.91 (brd, 4.9)
17	49.5	-	45.2	-	44.8	-
18	51.9	1.85 (m)	51.3	1.24 (m)	54.0	1.12 (dd, 12.9, 2.1)
19	33.1	2.35 (t, like, 12.4) 1.68 (m)	33.8	2.25 (t, like, 12.6) 1.58 (m)	34.0	2.50 (dd, 14.2, 12.9) 1.96 (dd, 14.2, 2.1)
20	36.7	-	36.9	-	49.2	-
21	32.3	2.03 (m) 1.35 (m)	33.2	2.04 (m) 1.28 (m)	30.8	2.12 (m) 1.89 (m)
22	28.6	1.84 (m) 1.60 (m)	31.8	1.72 (m) 1.45 (m)	32.8	1.84 (m) 1.32 (m)
23	28.7	0.99 (s)	28.3	1.06 (s)	28.3	1.05 (s)
24	16.8	0.78 (s)	16.7	0.83 (s)	16.7	0.83 (s)
25	16.7	0.91 (s)	16.7	0.89 (s)	16.7	0.89 (s)
26	18.2	1.08 (s)	18.8	1.14 (s)	18.8	1.13 (s)
27	19.8	1.40 (s)	20.0	1.25 (s)	20.1	1.27 (s)
28	181.0	-	78.6	3.50 (d, 7.6) 3.10 (d, 7.6)	78.4	3.48 (d, 7.6) 2.98 (d, 7.6)
29	28.3	0.92 (s)	28.5	0.92 (s)	24.3	0.97 (s)
30	66.5	3.55 (d, 11.1) 3.25 (d, 11.1)	66.5	3.55 (d, 10.9) 3.30 (d, 10.9)	209.2	9.40 (s)
1′	110.2	5.23 (brs)	105.9	4.39(d, 7.8)	106.0	4.40 (d, 7.0)
2′	89.6	4.21 (d, 5.2)	79.1	3.87 (m)	80.9	3.56 (m)
3′	75.8	4.26 (dd, 5.2, 2.0)	76.3	3.20 (m)	78.0	3.53 (m)
4′	83.9	4.19 (m)	70.3	3.83 (m)	71.1	3.49 (m)
5′	62.5	3.83 (dd, 11.6, 4.9) 3.75 (dd, 11.6, 6.4)	76.2	3.47 (m)	78.4	3.47 (m)
6′	-	-	62.3	3.72 (overlap) 3.70 (overlap)	63.0	3.81 (dd, 11.9, 2.0) 3.60 (dd, 11.9, 5.8)
1″	104.2	4.44 (d, 7.8)	104.6	4.65 (d, 7.7)	104.6	4.65 (d, 7.7)
2″	75.0	3.18 (m)	75.4	3.66 (m)	76.2	3.20 (m)
3″	78.0	3.25 (m)	78.2	3.22 (m)	77.9	3.33 (m)
4″	71.3	3.36 (m)	71.9	3.21 (m)	71.9	3.21 (m)
5″	77.9	3.34 (m)	77.9	3.34 (m)	66.5	3.84 (overlap) 3.17 (overlap)
6″	62.4	3.85 (dd, 12.0, 2.5) 3.72 (dd, 12.0, 5.0)	63.1	3.81 (dd, 11.9, 2.2) 3.61 (dd, 11.9, 5.9)	-	-

**Table 2:** Cytotoxic activities of compounds **1**–**7** against three human tumor cell lines (HeLa, HepG2 and U87 MG) (mean ± SD, *n* = 3).

Compounds	IC_50_ (Mean ± SD μM)		
	HeLa	HepG2	U87 MG
1	3.2 ± 0.7	8.8 ± 2.2	5.7 ± 1.8
2	4.4 ± 1.1	21.9 ± 6.1	6.8 ± 2.2
3	2.2 ± 0.6	33.6 ± 6.8	7.7 ± 0.9
4	6.8 ± 1.2	7.9 ± 2.2	6.6 ± 1.6
5	9.5 ± 1.8	14.4 ± 2.1	2.3 ± 0.4
6	5.4 ± 0.9	8.7 ± 1.2	6.8 ± 1.1
7	5.2 ± 1.3	38.2 ± 6.6	5.4 ± 0.9
Cisplatin *^a^*	9.8 ± 0.4	12.1 ± 0.3	16.7± 0.8

*^a^* Positive control.

## Data Availability

Not available.

## Supplementary Materials

The spectroscopic data for compounds **1–7** (Figures S1–S50) are available as Supporting Information. Table S1: ^13^C-NMR (100 MHz) Data of **4**-**7** in CD_3_OD.

## Sample Availability

Samples of all the isolated compounds are available from the authors.
