# Supplementary material for: Cytotoxic 13,28 Epoxy Bridged Oleanane-Type Triterpenoid Saponins from the Roots of Ardisia crispa
Source: Molecules. 2022 Feb 4;27(3):1061. doi: 10.3390/molecules27031061 (PMC8838445; doi:10.3390/molecules27031061)
Supplement: Supplementary file 1 [file molecules-27-01061-s001.zip › molecules-1584297- Supplementary- corrected.pdf]

Supporting Information

Cytotoxic 13,28 epoxy bridged oleanane-type triterpenoid saponins from the roots of *Ardisia crispa*

Xin Yin[1], Rui-hang Hu[1], Yong-qiang Zhou, Wei-qian Zhu and Ying Zhou[*]

College of Pharmacy, Guizhou University of Traditional Chinese Medicine, Guiyang 550025, P. R. China

Correspondence

Professor Ying Zhou
College of Pharmacy, Guizhou University of Traditional Chinese Medicine
Dongqing Road, Huaxi District, Guiyang 550025, P.R. China
E-mail: Yingzhou71@sina.com

[1] These authors contributed equally to this work

Legends for Supplementary Figures

Figure S1: ^{1}H-NMR Spectrum of Compound **1**

Figure S2-S4 ^{1}H-NMR extended spectrum of Compound **1**

Figure S5: ^{13}C NMR spectrum of Compound **1**

Figure S6-S7 ^{13}C NMR extended spectrum of Compound **1**

Figure S8: DEPT-135 spectrum of Compound **1**

Figure S9: HSQC spectrum of Compound **1**

Figure S10: ^{1}H-^{1}H COSY spectrum of Compound **1**

Figure S11: HMBC spectrum of Compound **1**

Figure S12: NOESY spectrum of Compound **1**

Figure S13: HR-ESI-MS spectrum of Compound **1**

Figure S14: ESI-MS spectrum of Compound **1**

Figure S15: ^{1}H-NMR Spectrum of Compound **2**

Figure S16-S19: ^{1}H-NMR extended spectrum of Compound **2**

Figure S20: ^{13}C NMR spectrum of Compound **2**

Figure S21-22: ^{13}C NMR extended spectrum of Compound **2**

Figure S23: DEPT-135 spectrum of Compound **2**

Figure S24: HSQC spectrum of Compound **2**

Figure S25: ^{1}H-^{1}H COSY spectrum of Compound **2**

Figure S26: HMBC spectrum of Compound **2**

Figure S27: NOESY spectrum of Compound **2**

Figure S28: HR-ESI-MS spectrum of Compound **2**

Figure S29: ESI-MS spectrum of Compound **2**

Figure S30: ^{1}H-NMR Spectrum of Compound **3**

Figure S31-32: ^{1}H-NMR extended spectrum of Compound **3**

Figure S33: ^{13}C NMR spectrum of Compound **3**

Figure S34-35: ^{13}C NMR extended spectrum of Compound **3**

Figure S36: DEPT-135 spectrum of Compound **3**

Figure S37: HSQC spectrum of Compound **3**

Figure S38: ^{1}H-^{1}H COSY spectrum of Compound **3**

Figure S39: HMBC spectrum of Compound **3**

Figure S40: NOESY spectrum of Compound **3**

Figure S41: HR-ESI-MS spectrum of Compound **3**

Figure S42: ESI-MS spectrum of Compound **3**

Figure S43: ^{1}H-NMR spectrum of Compound **4**

Figure S44: ^{13}C NMR spectrum of Compound **4**

Figure S45: ^{1}H-NMR spectrum of Compound **5**

Figure S46: ^{13}C NMR spectrum of Compound **5**

Figure S47: ^{1}H-NMR spectrum of Compound **6**

Figure S48: ^{13}C NMR spectrum of Compound **6**

Figure S49: ^{1}H-NMR spectrum of Compound **7**

Figure S50: ^{13}C NMR spectrum of Compound **7**

Legends for Supplementary Table

Table S1 ^{13}C-NMR (100 MHz) Data of **4-7** in CD_3OD

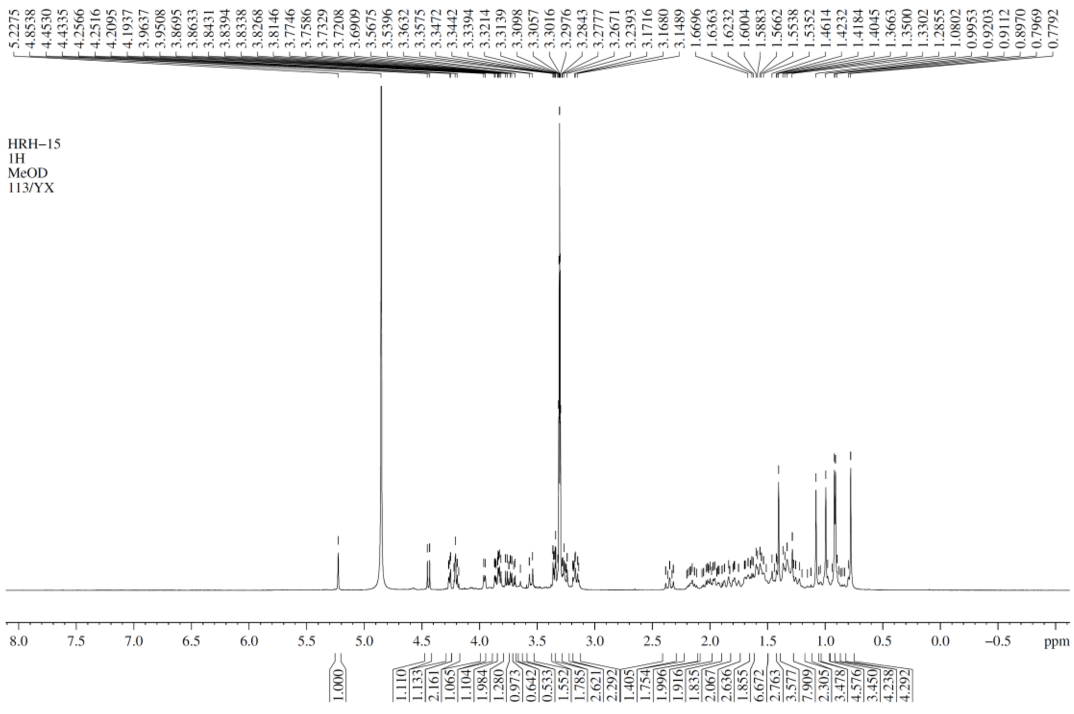

Figure S1: ^{1}H-NMR spectrum of Compound **1**

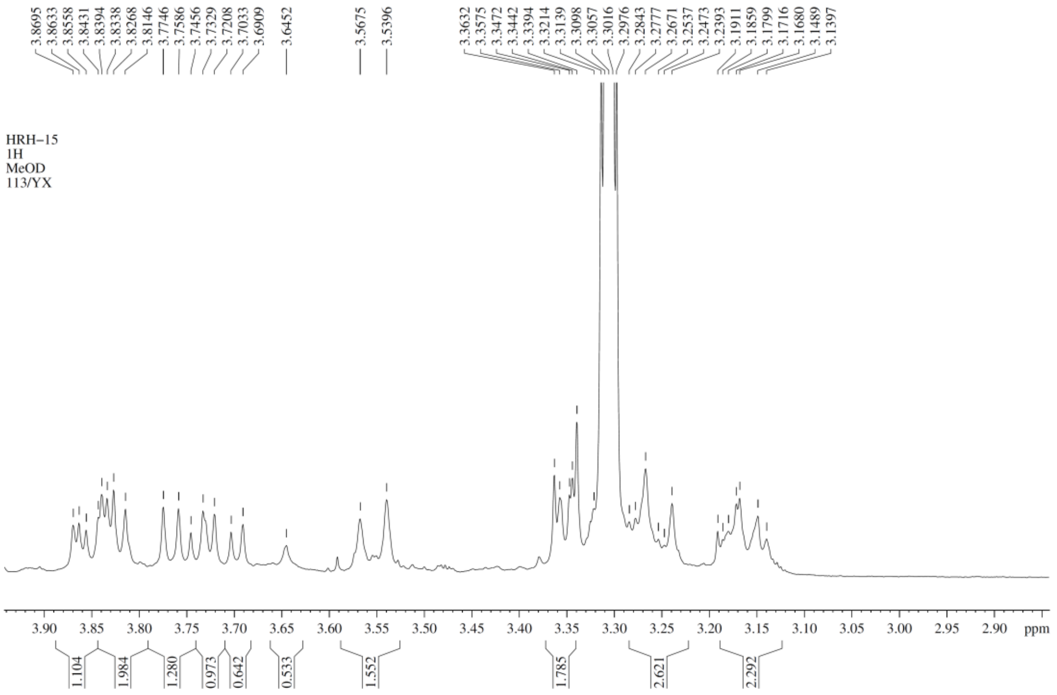

Figure S2: ^{1}H-NMR extended spectrum of Compound **1**

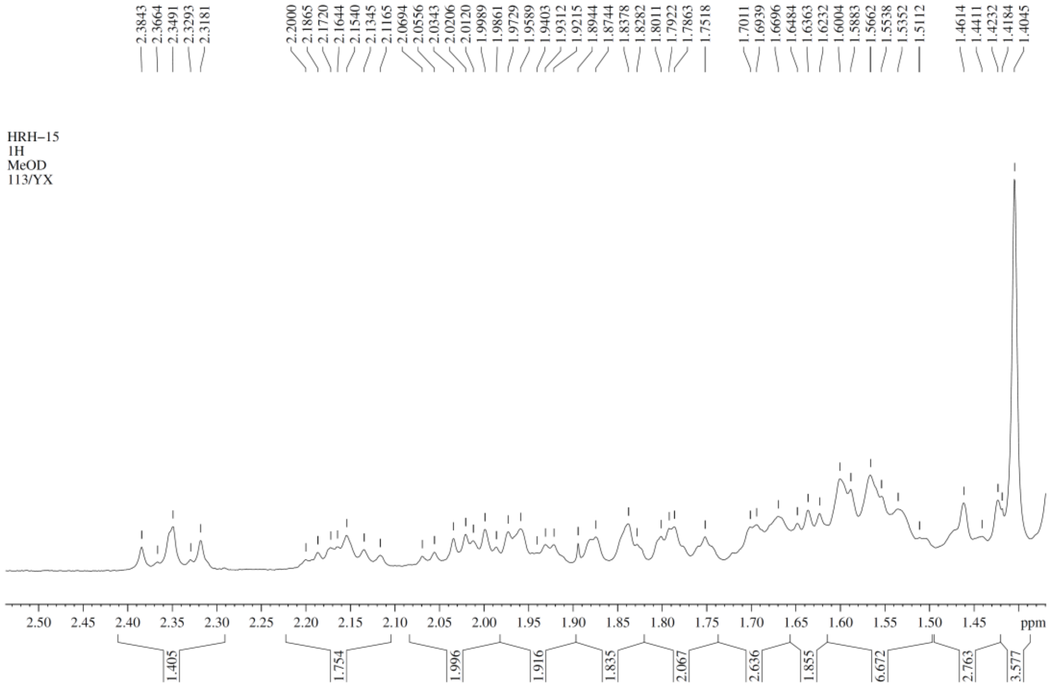

Figure S3: ^{1}H-NMR extended spectrum of Compound **1**

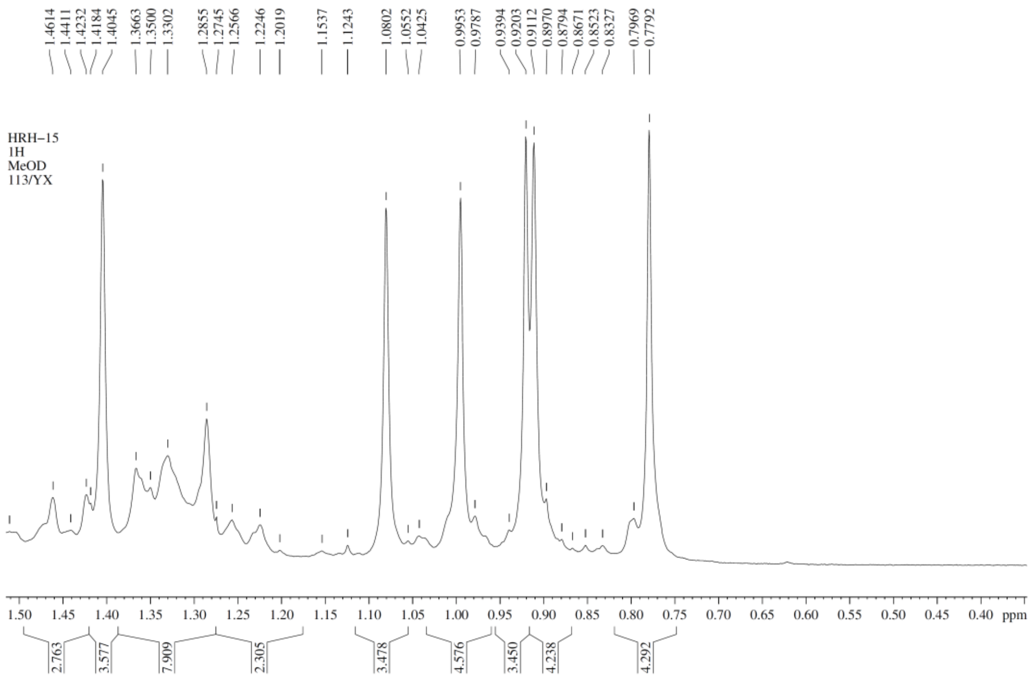

Figure S4: ^{1}H-NMR extended spectrum of Compound **1**

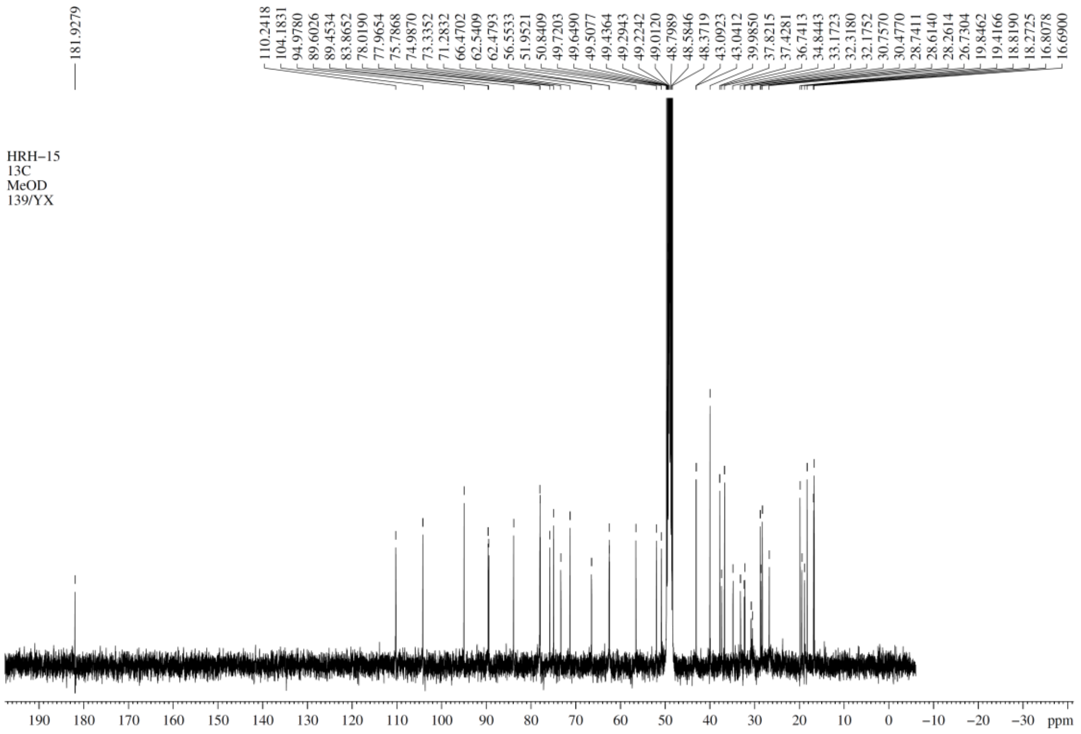

Figure S5: ^{13}C NMR spectrum of Compound **1**

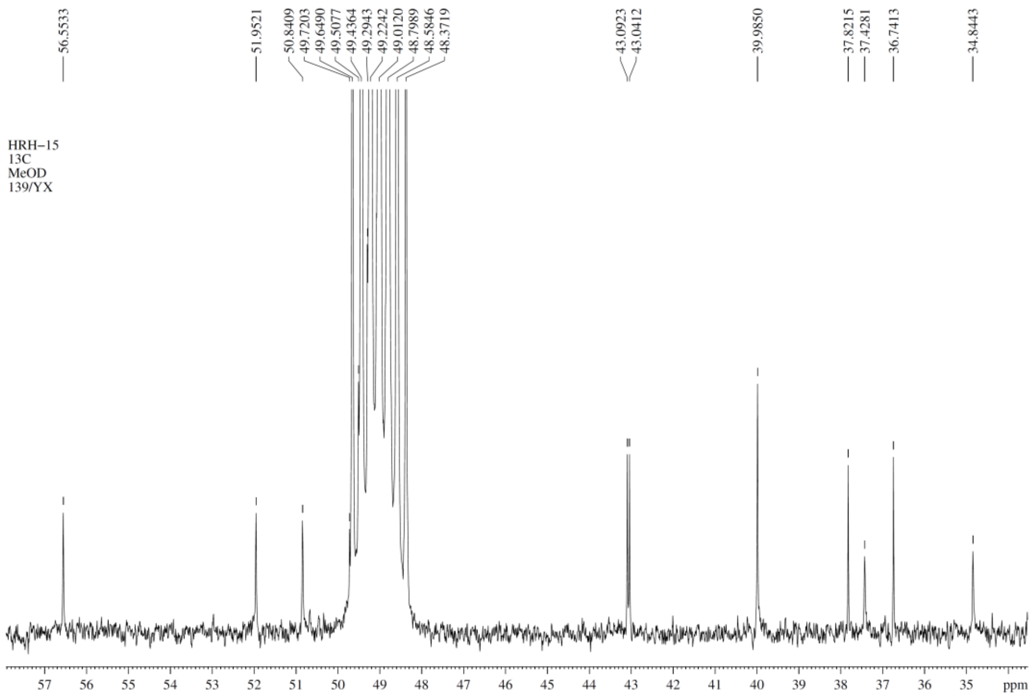

Figure S6: ^{13}C NMR extended spectrum of Compound **1**

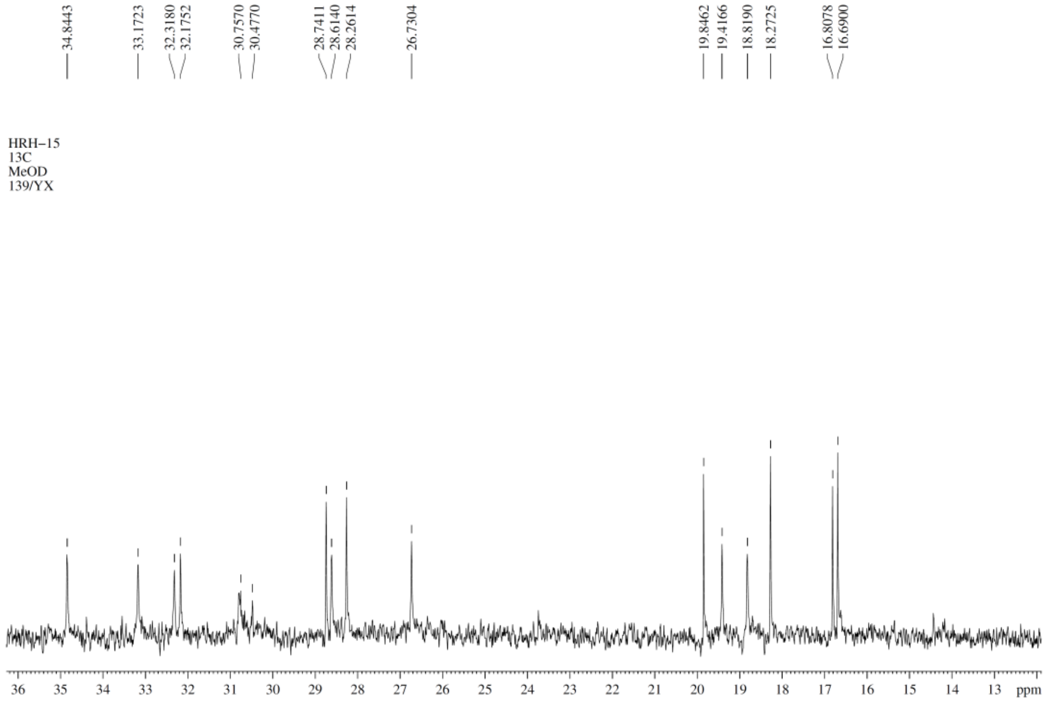

Figure S7: ^{13}C NMR extended spectrum of Compound **1**

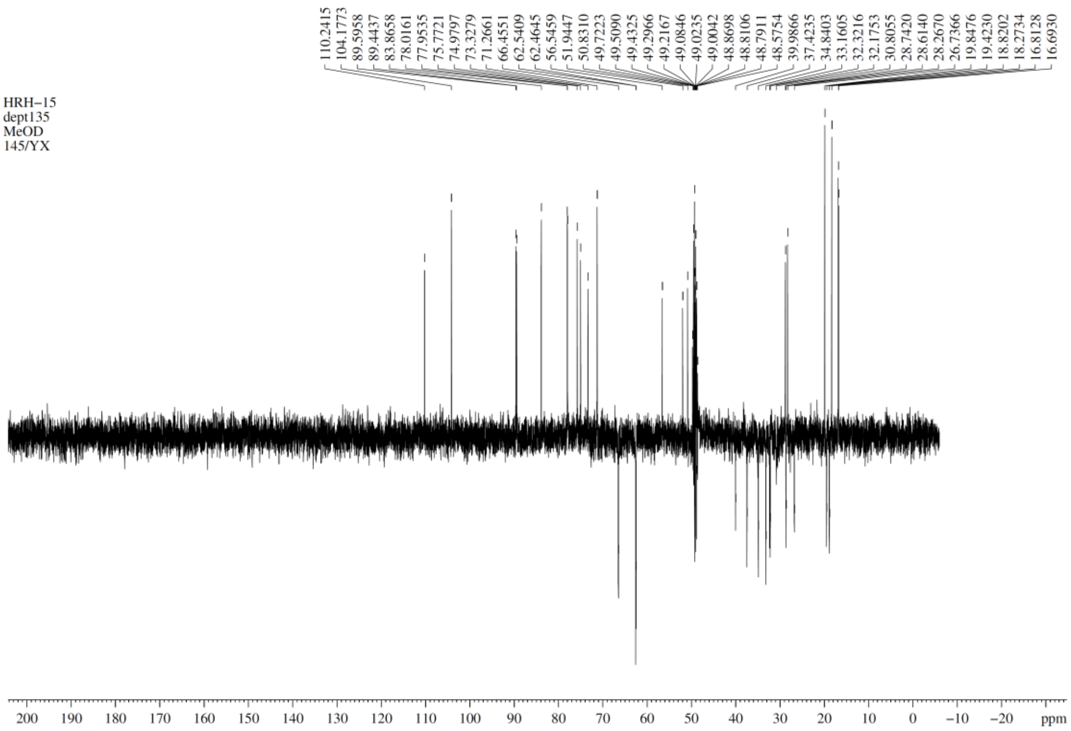

Figure S8: DEPT-135 spectrum of Compound **1**

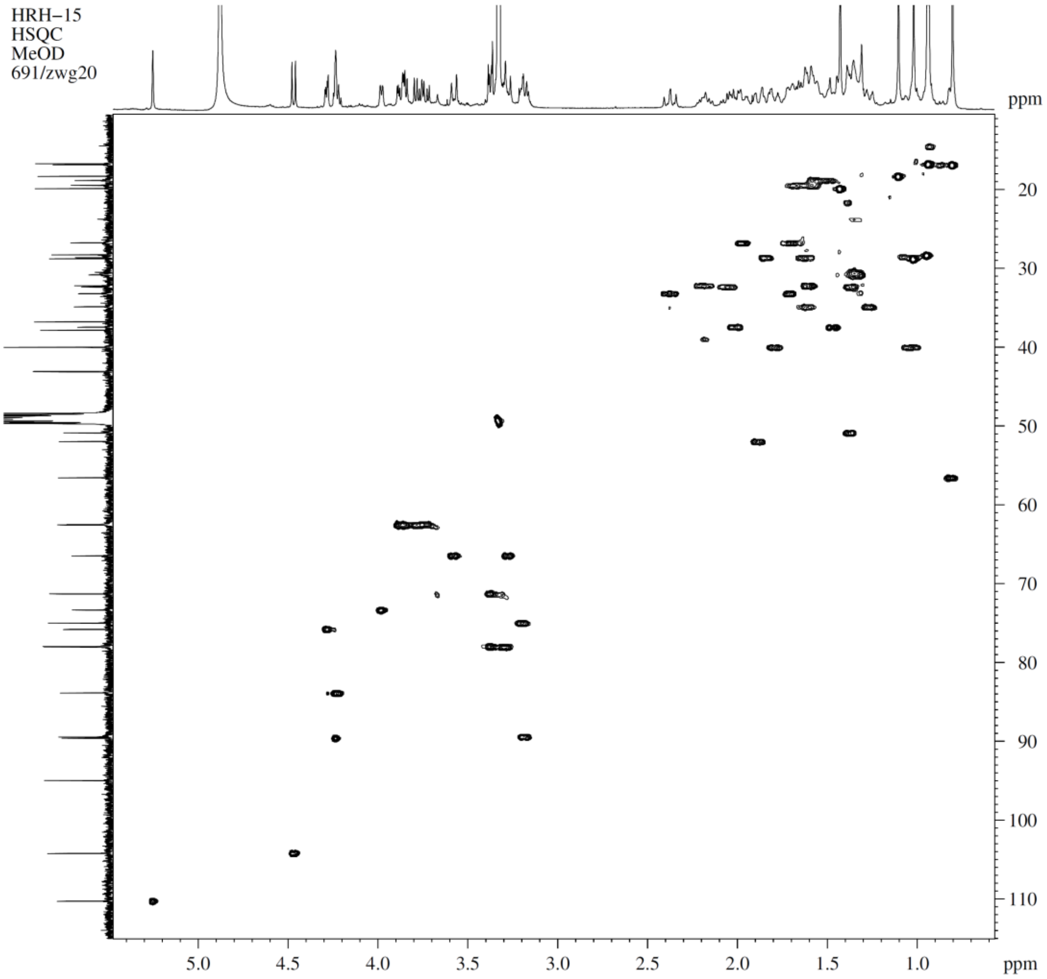

Figure S9: HSQC spectrum of Compound **1**

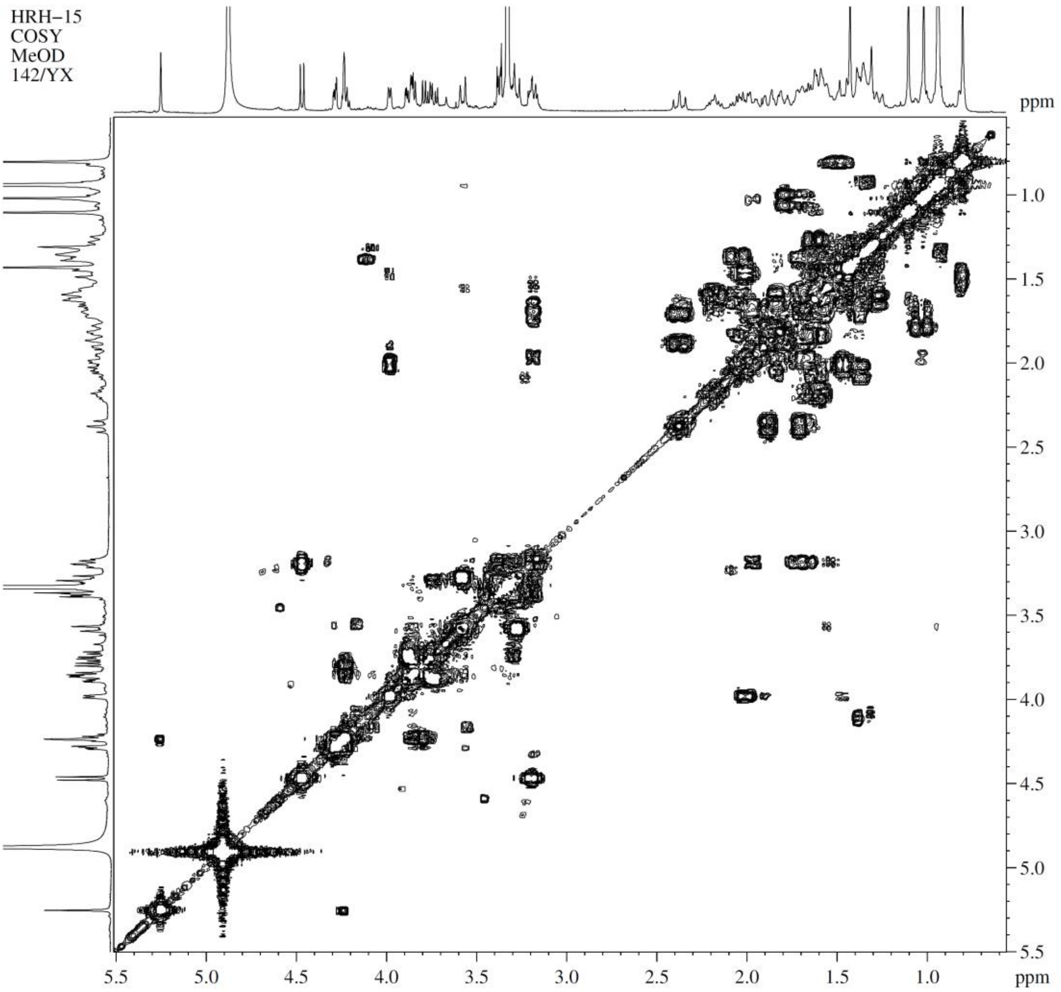

Figure S10: ^{1}H-^{1}H COSY spectrum of Compound **1**

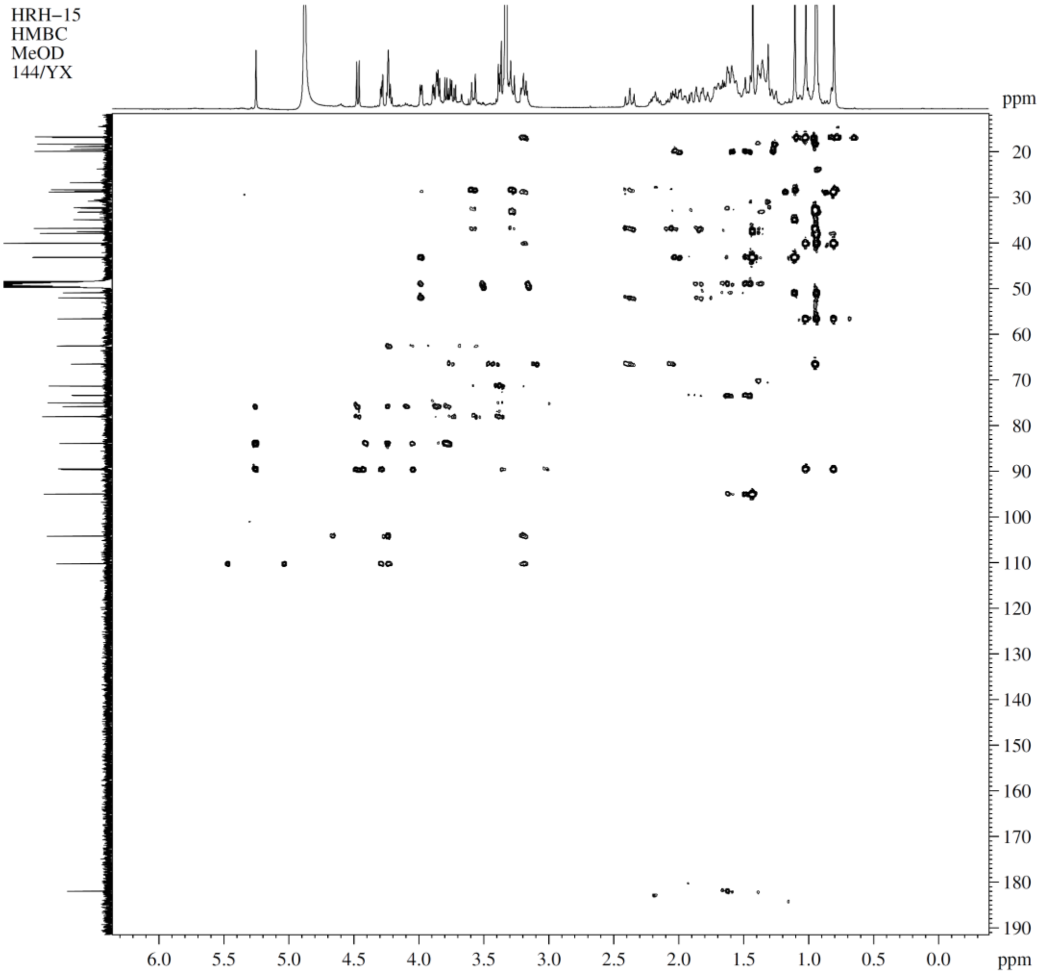

Figure S11: HMBC spectrum of Compound **1**

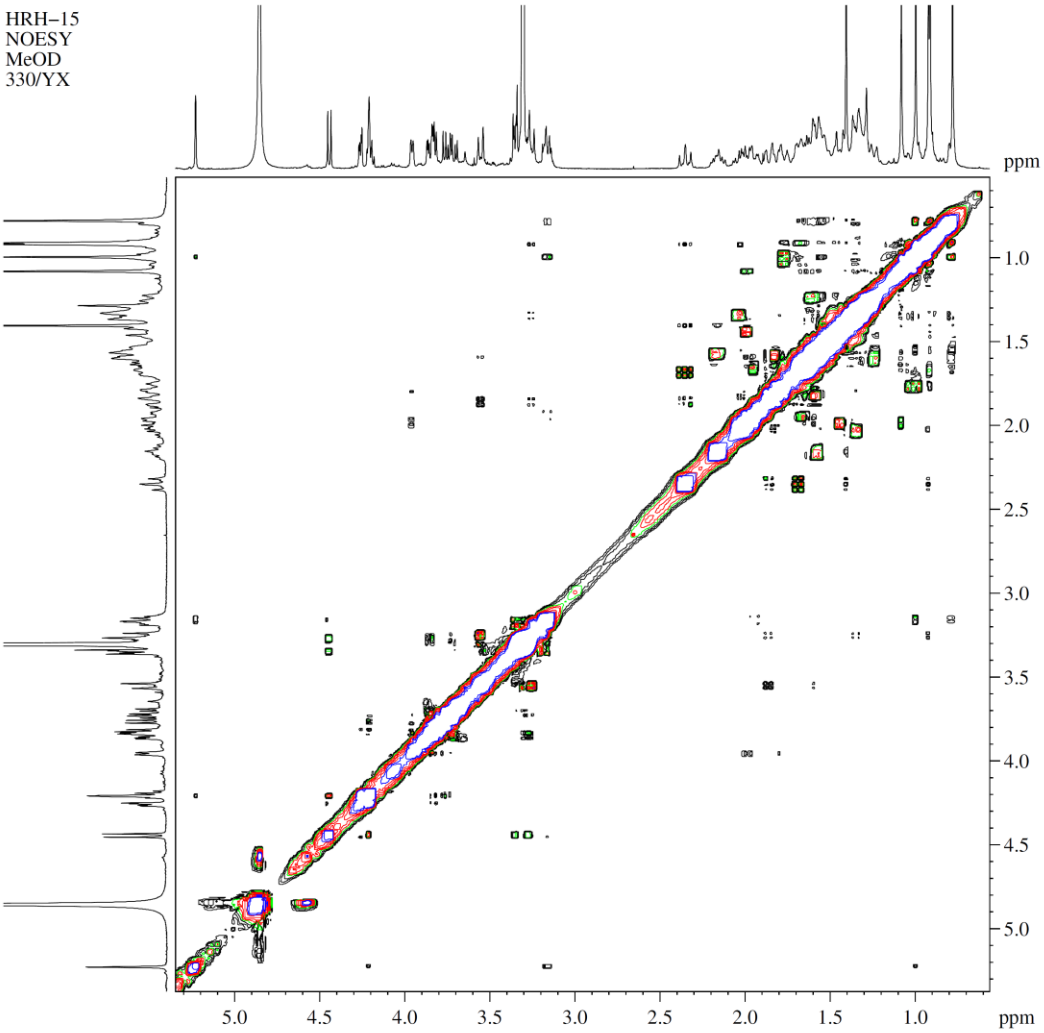

Figure S12: NOESY spectrum of Compound **1**

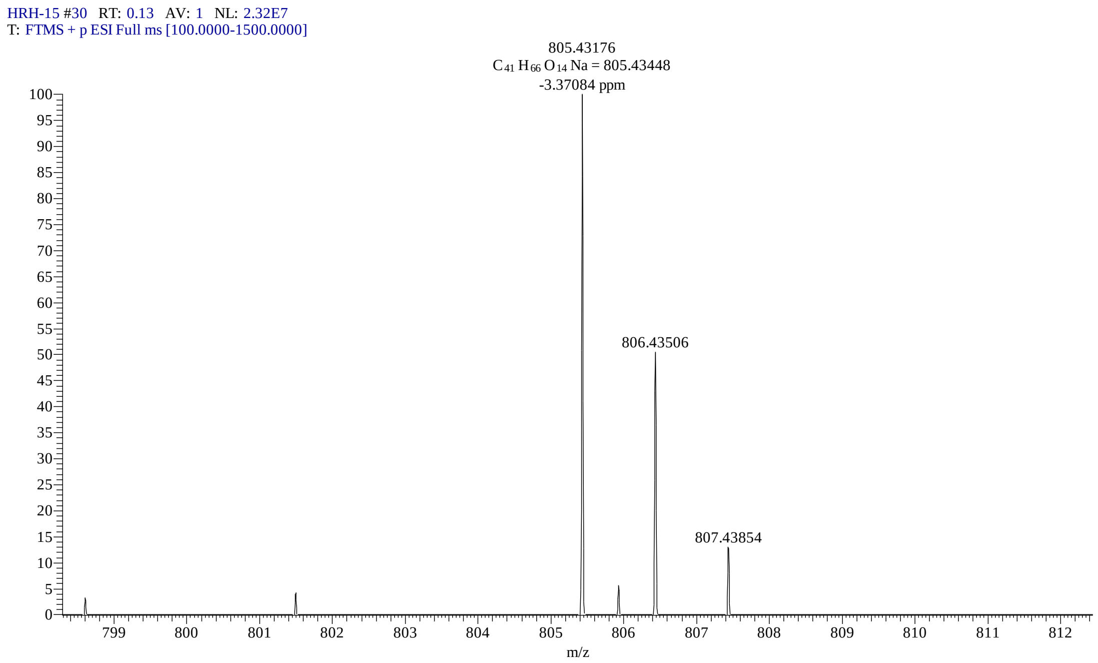

Figure S13: HR-ESI-MS spectrum of Compound **1**

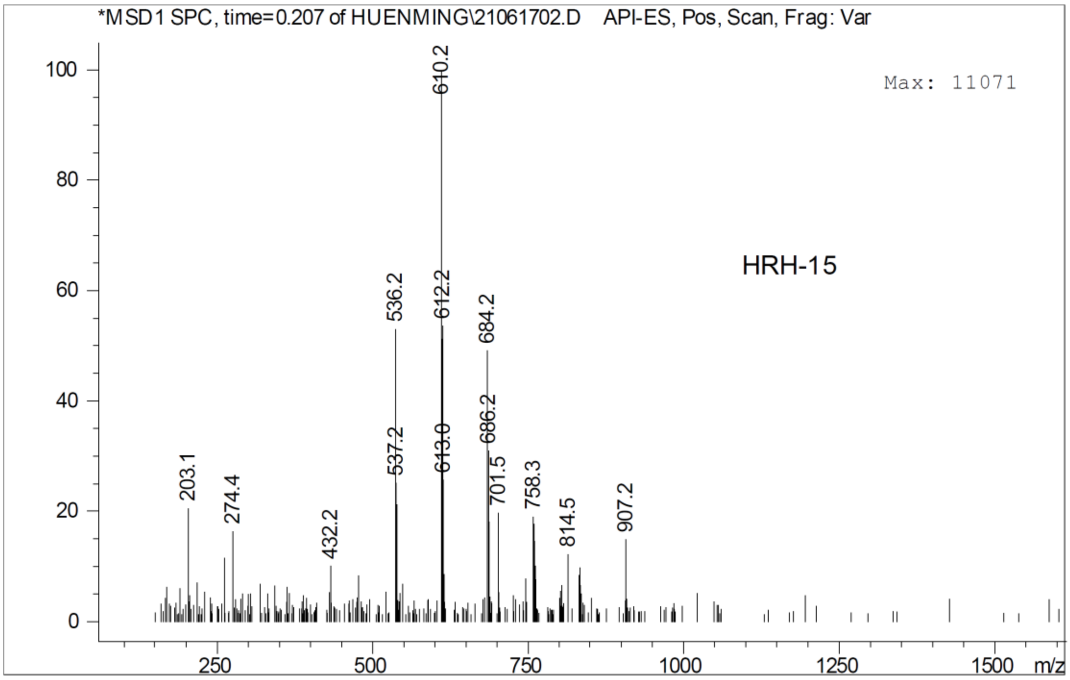

Figure S14: ESI-MS spectrum of Compound **1**

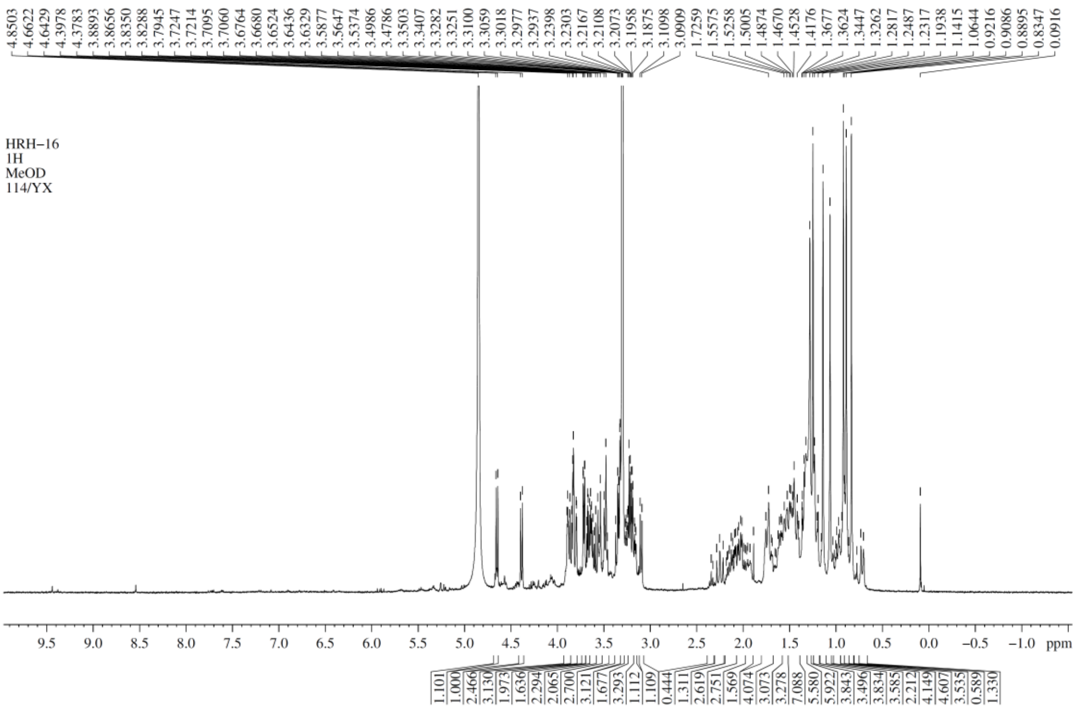

Figure S15: ^{1}H-NMR spectrum of Compound **2**

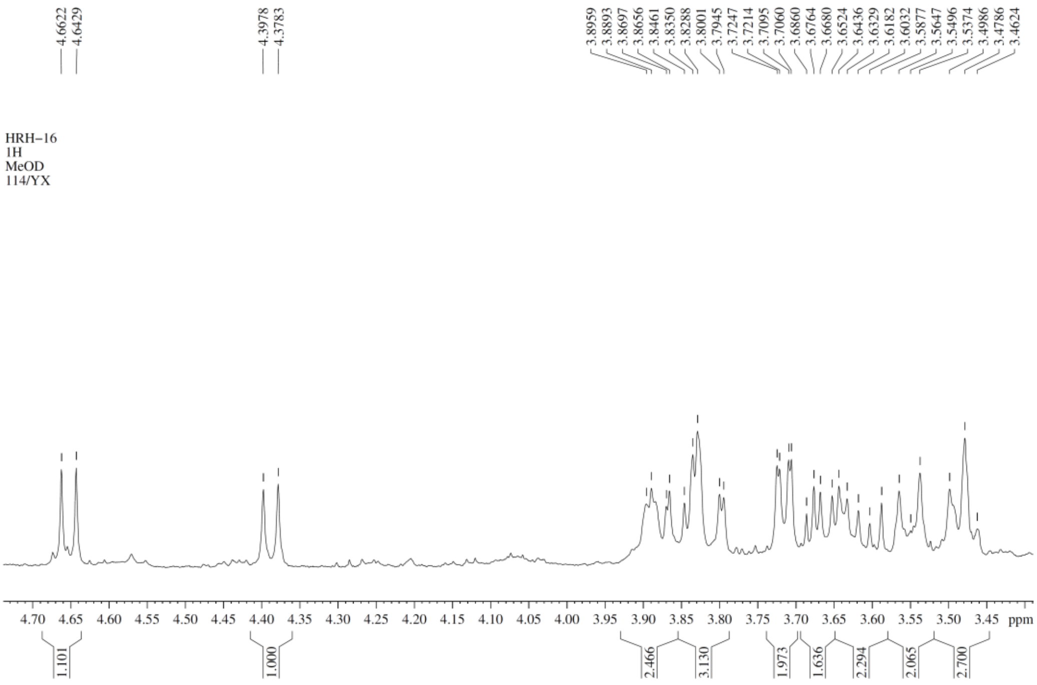

Figure S16: ^{1}H-NMR extended spectrum of Compound **2**

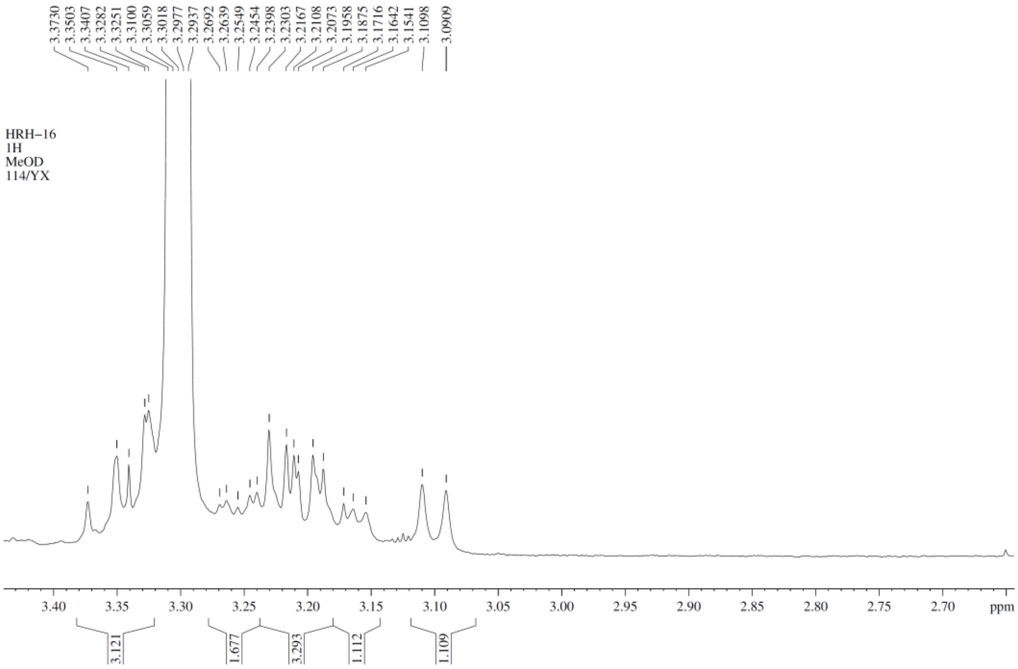

Figure S17: ^{1}H-NMR extended spectrum of Compound **2**

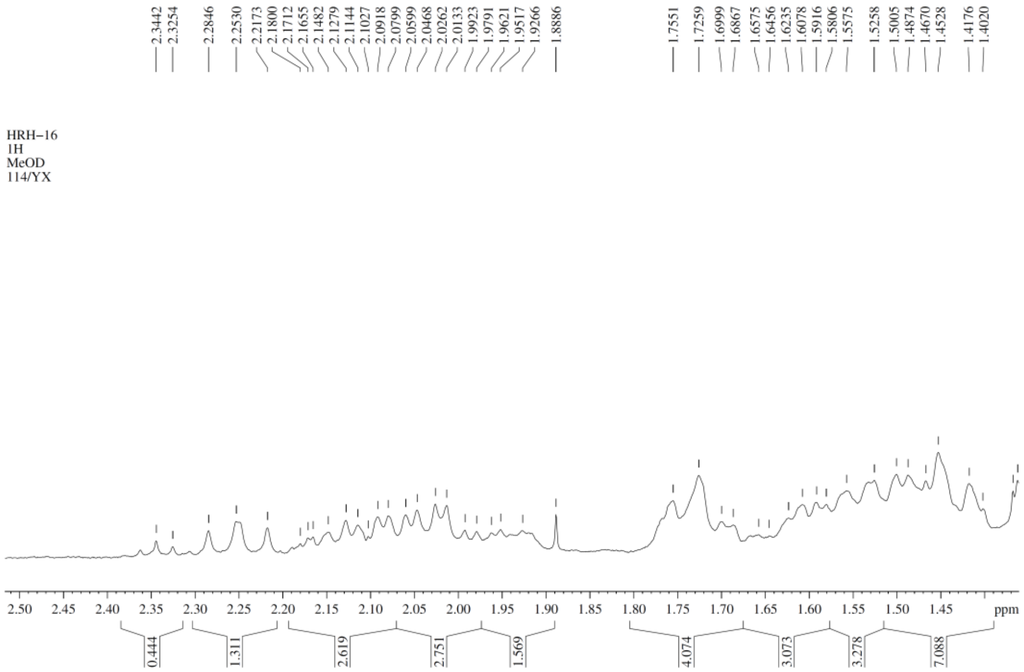

Figure S18: ^{1}H-NMR extended spectrum of Compound **2**

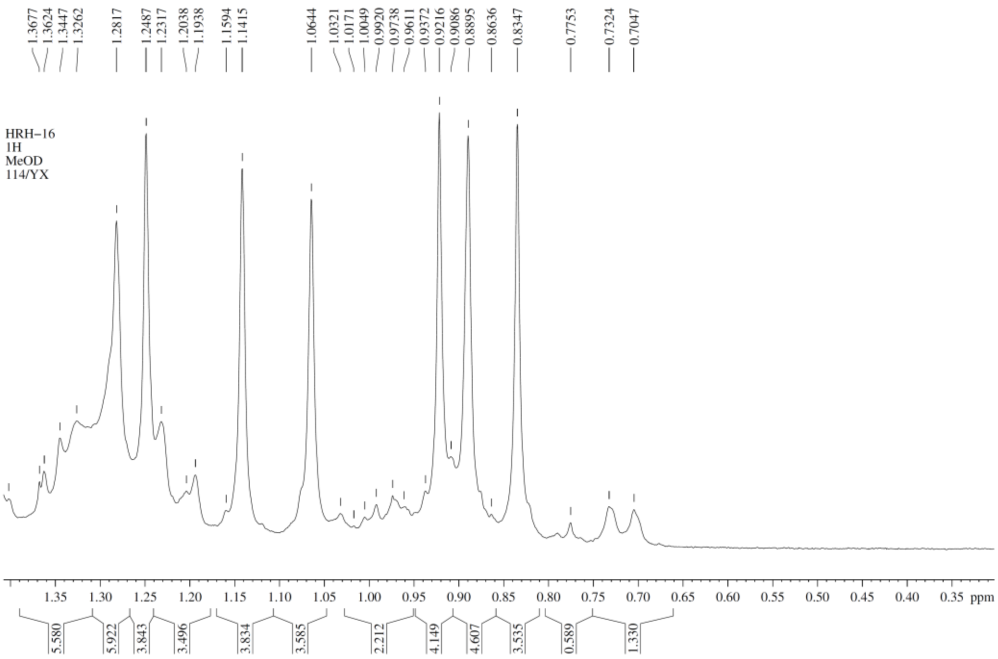

Figure S19: ^{1}H-NMR extended spectrum of Compound **2**

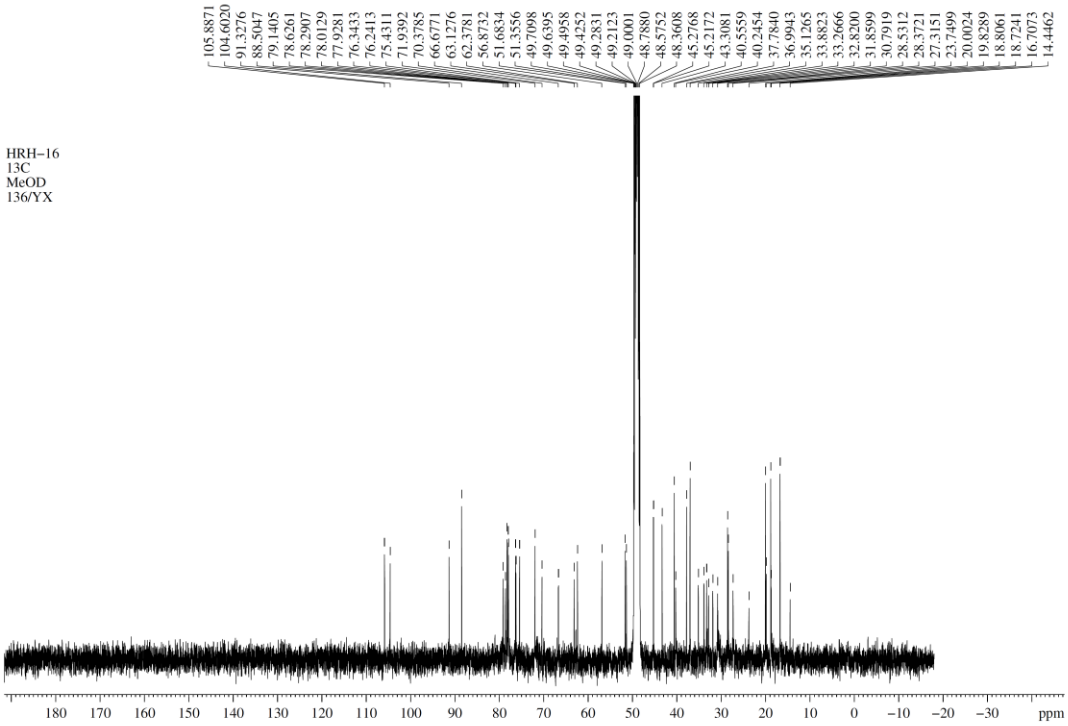

Figure S20: ^{13}C NMR spectrum of Compound **2**

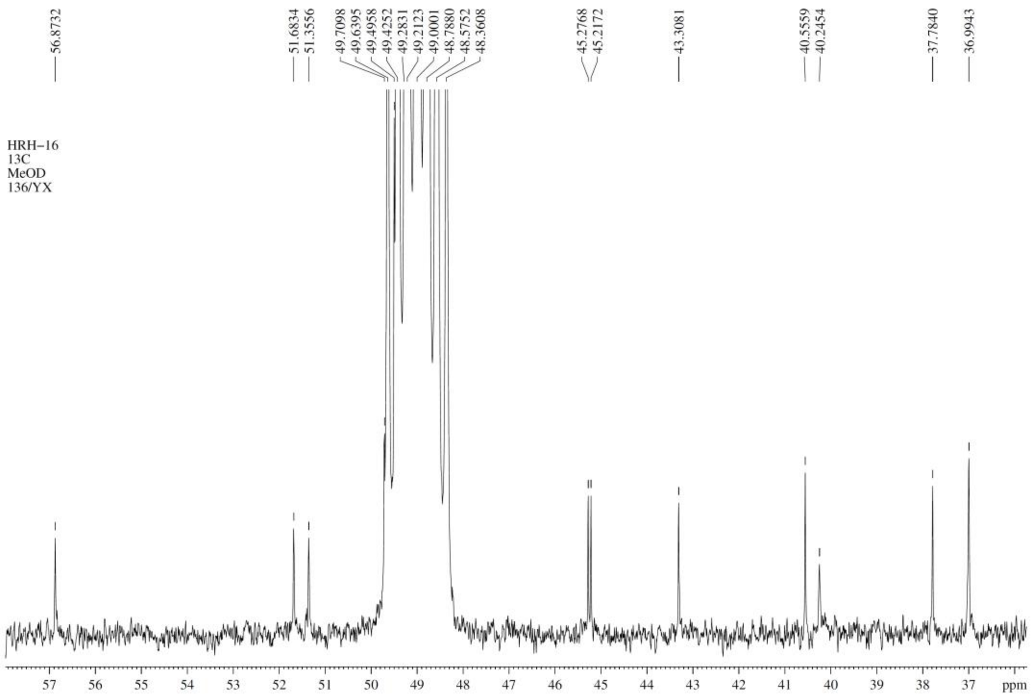

Figure S21: ^{13}C NMR extended spectrum of Compound **2**

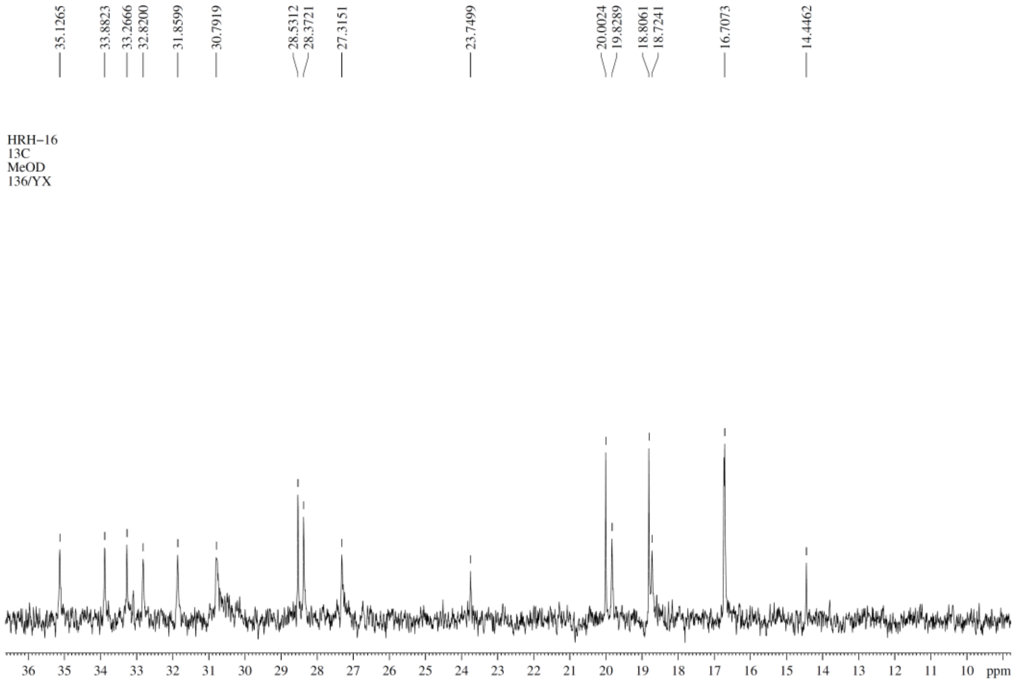

Figure S22: ^{13}C NMR extended spectrum of Compound **2**

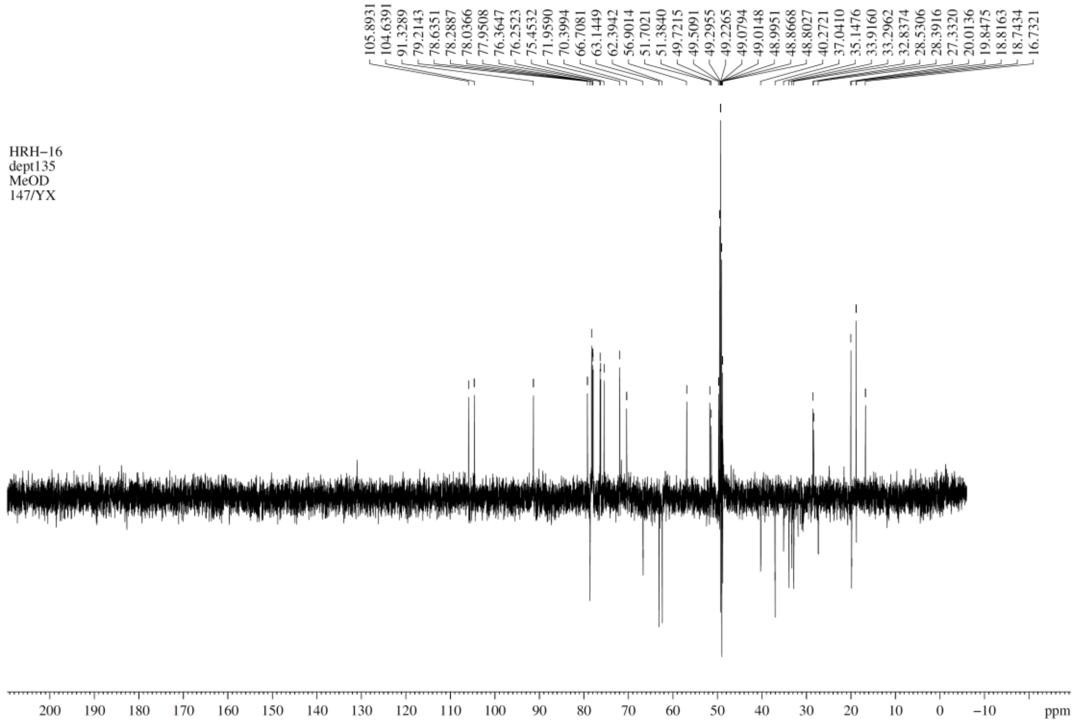

Figure S23: DEPT-135 spectrum of Compound **2**

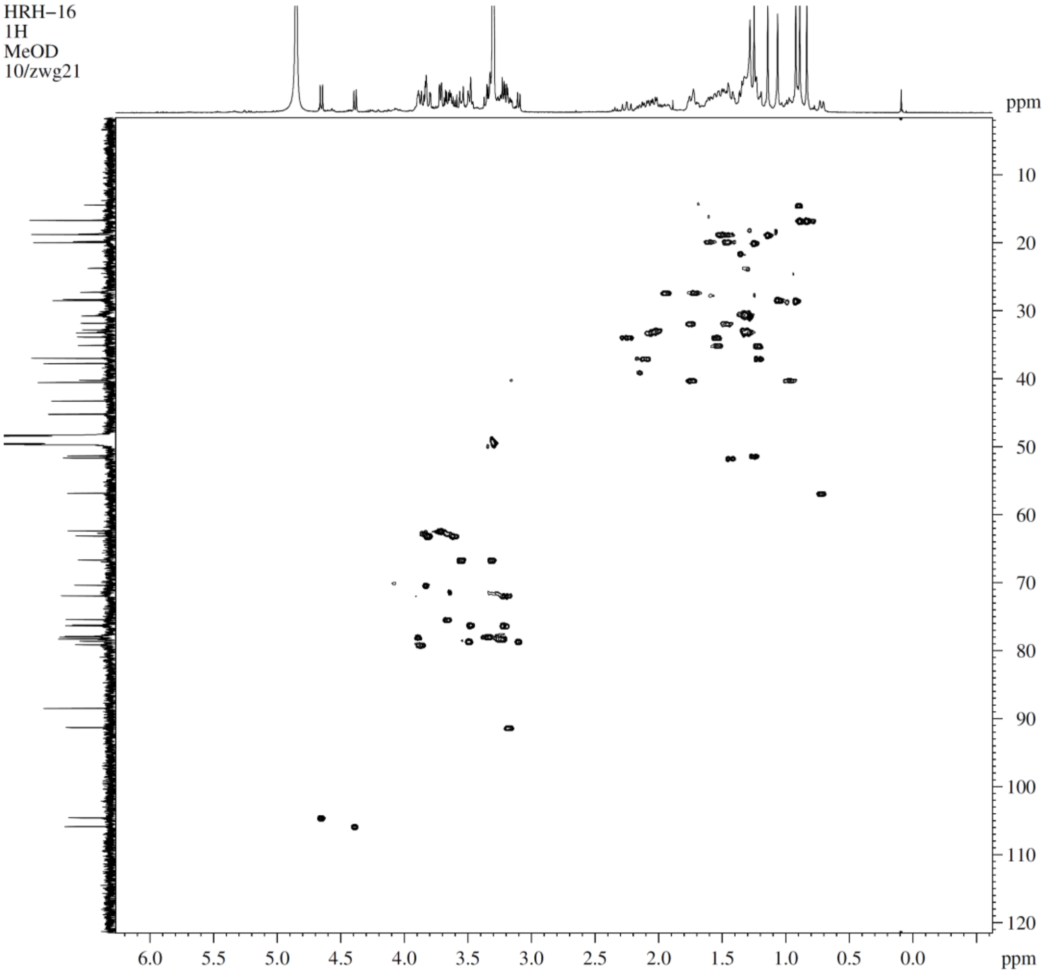

Figure S24: HSQC spectrum of Compound **2**

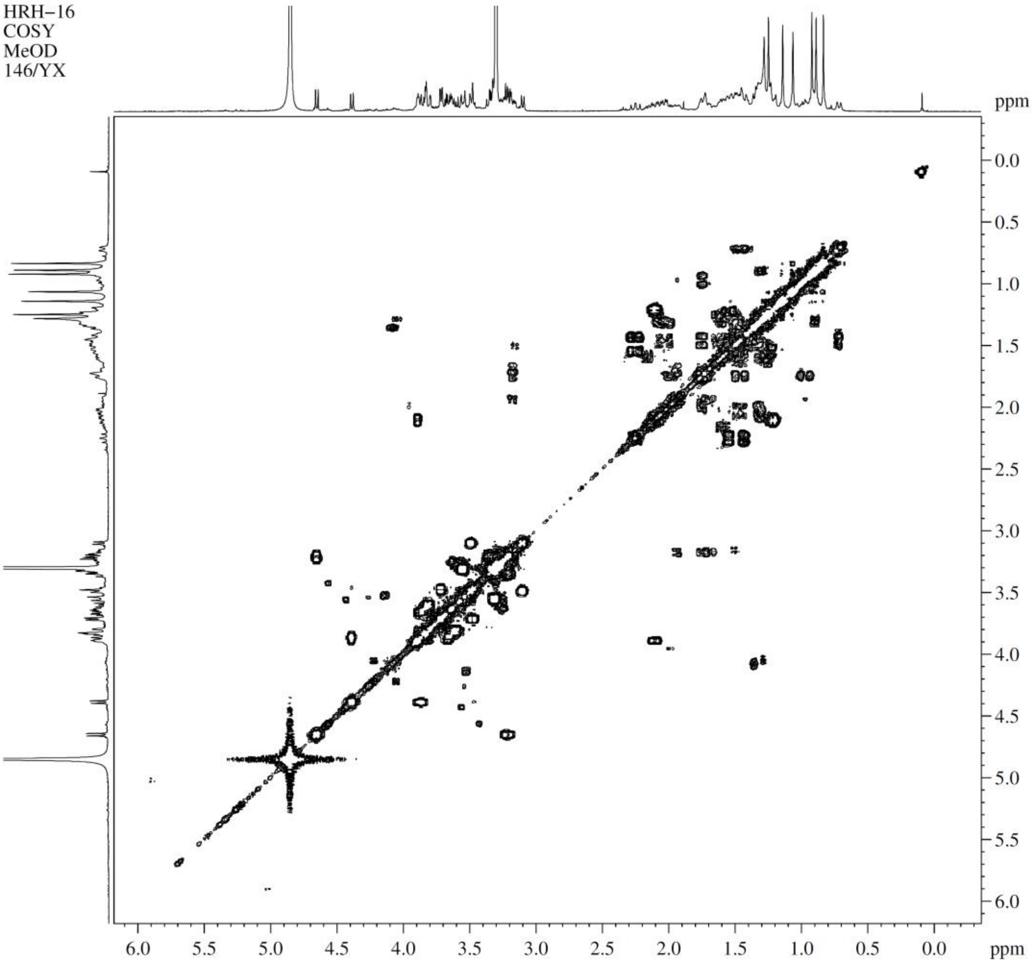

Figure S25: ^{1}H-^{1}H COSY spectrum of Compound **2**

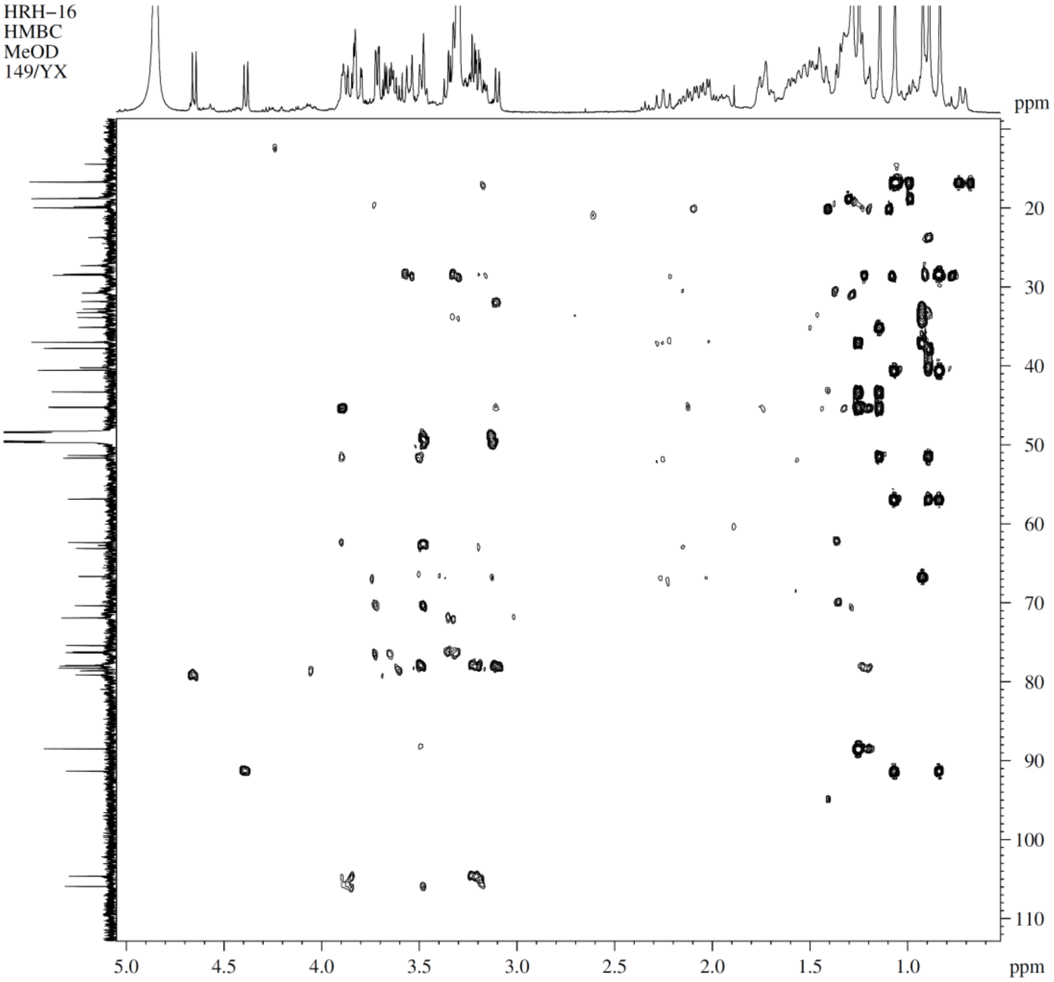

Figure S26: HMBC spectrum of Compound **2**

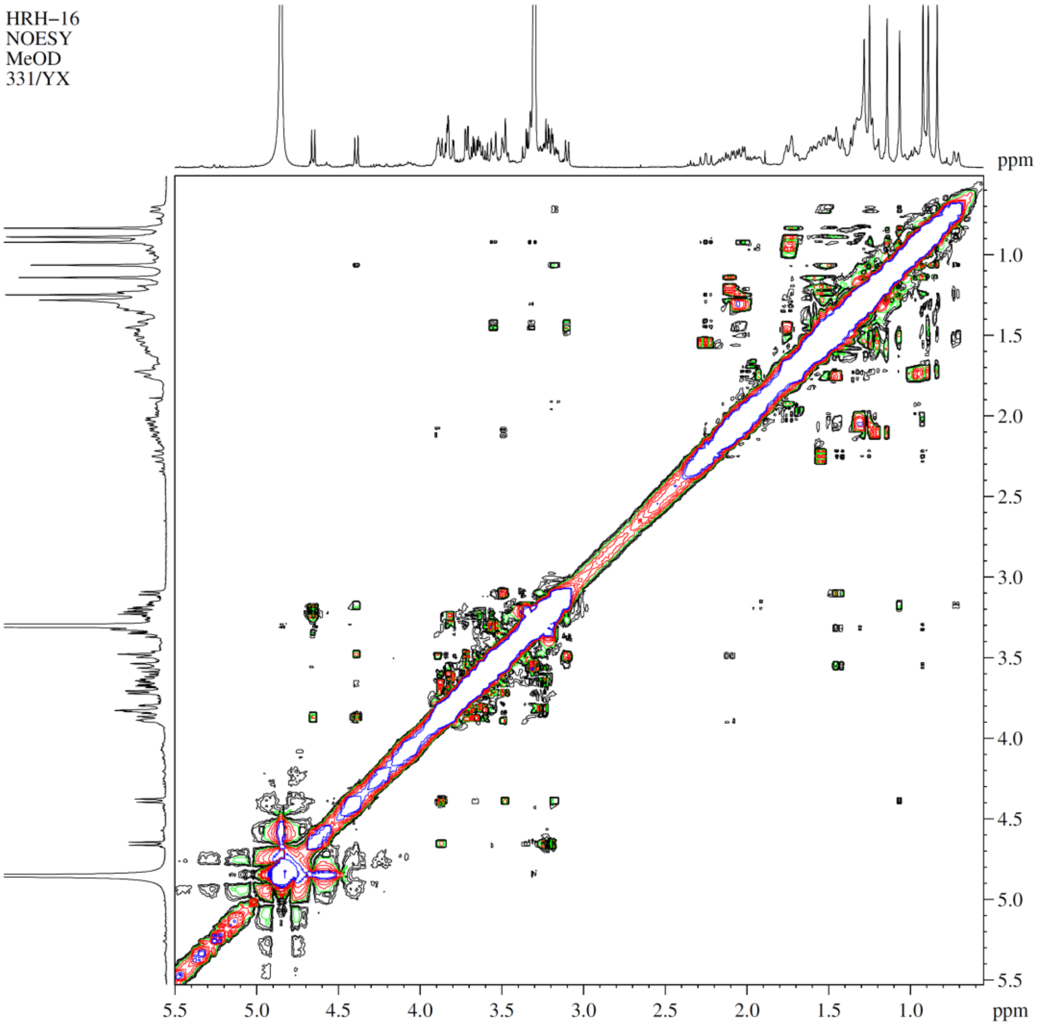

Figure S27: NOESY spectrum of Compound **2**

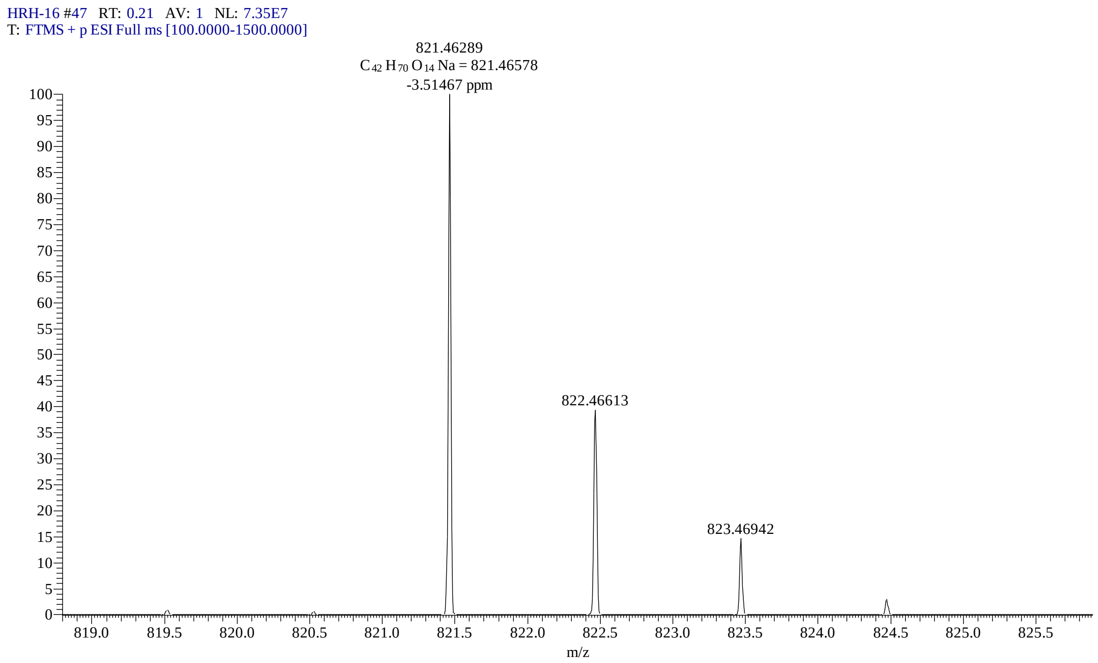

Figure S28: HR-ESI-MS spectrum of Compound **2**

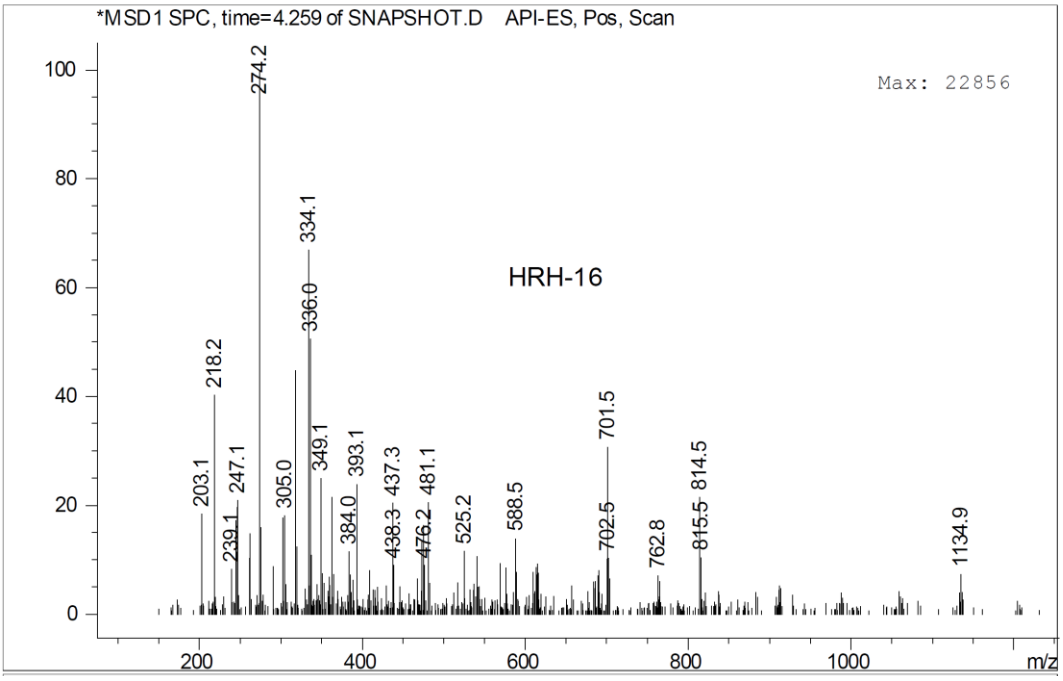

Figure S29: ESI-MS spectrum of Compound **2**

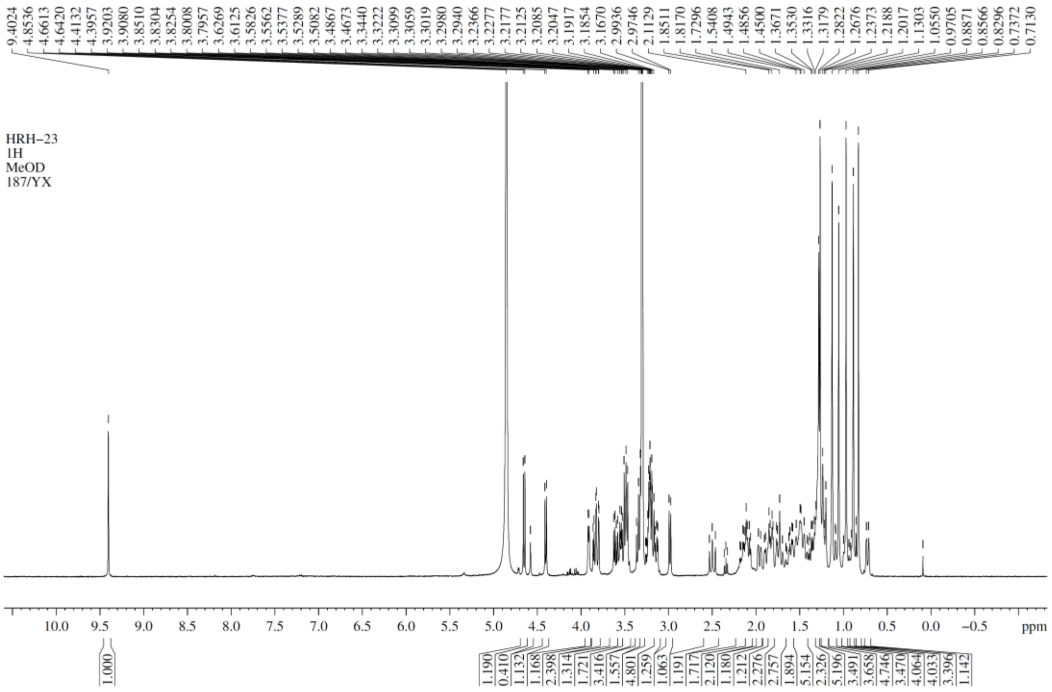

Figure S30: ^{1}H-NMR spectrum of Compound **3**

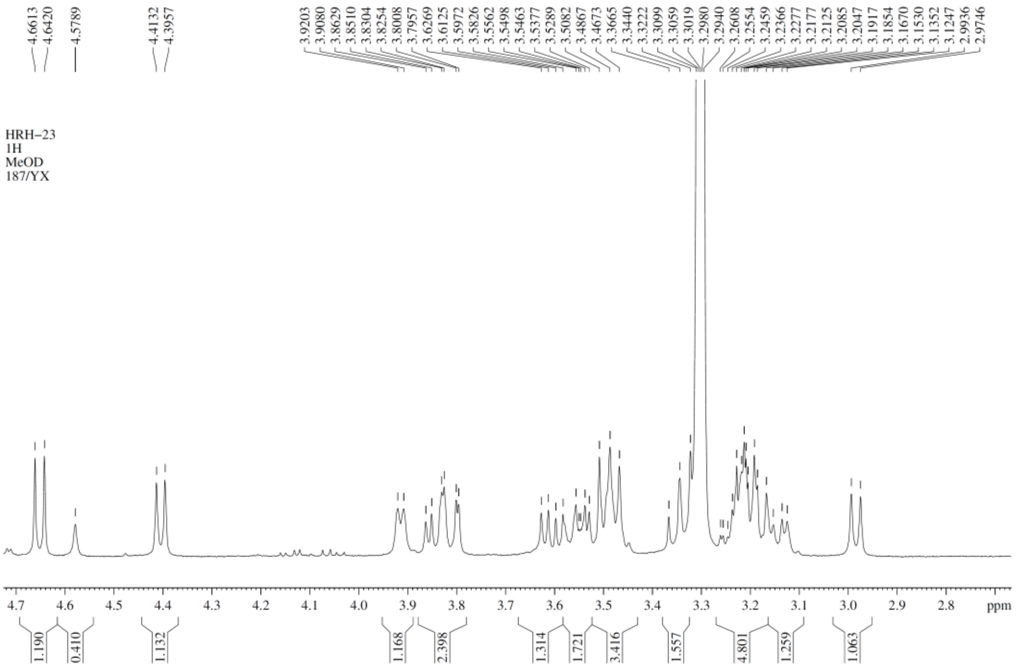

Figure S31: ^{1}H-NMR extended spectrum of Compound **3**

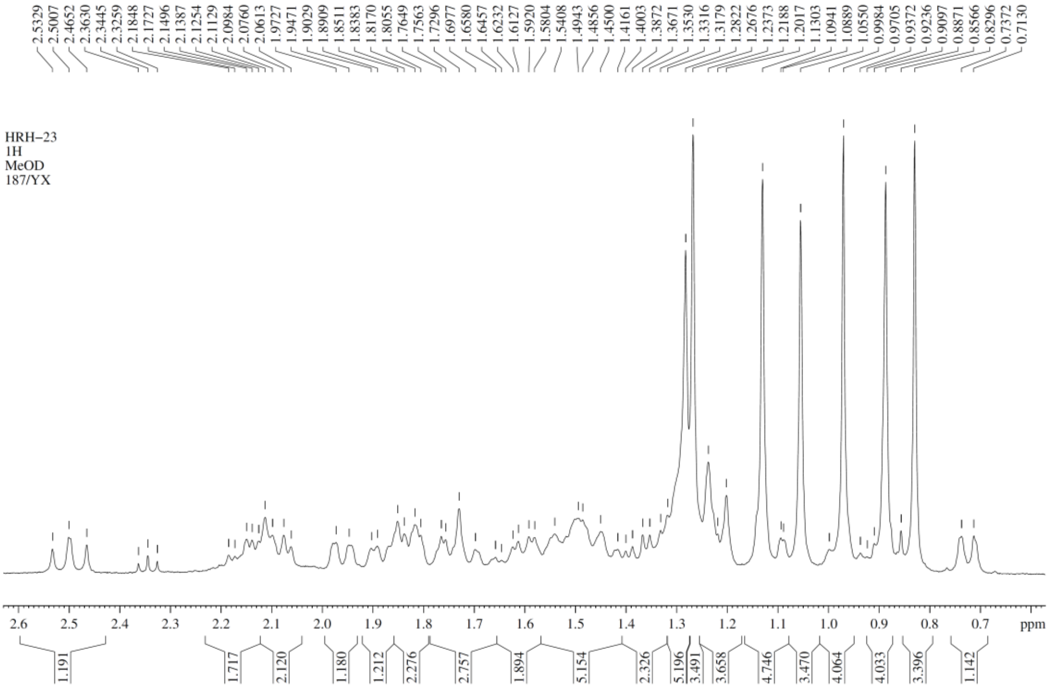

Figure S32: ^{1}H-NMR extended spectrum of Compound **3**

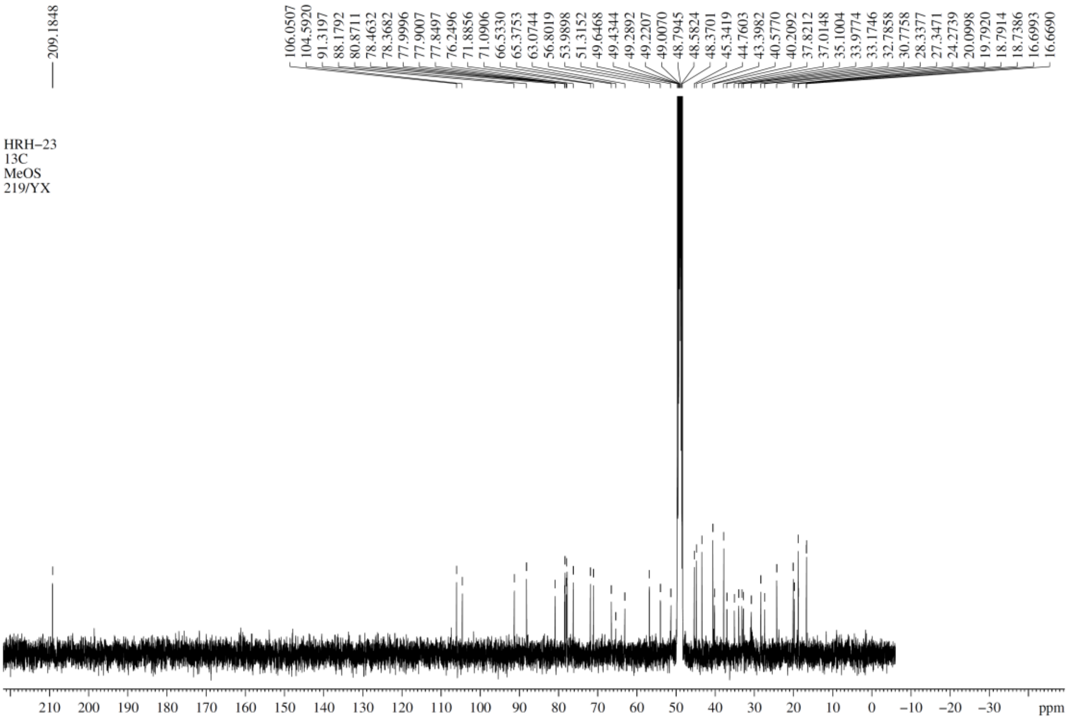

Figure S33: ^{13}C NMR spectrum of Compound **3**

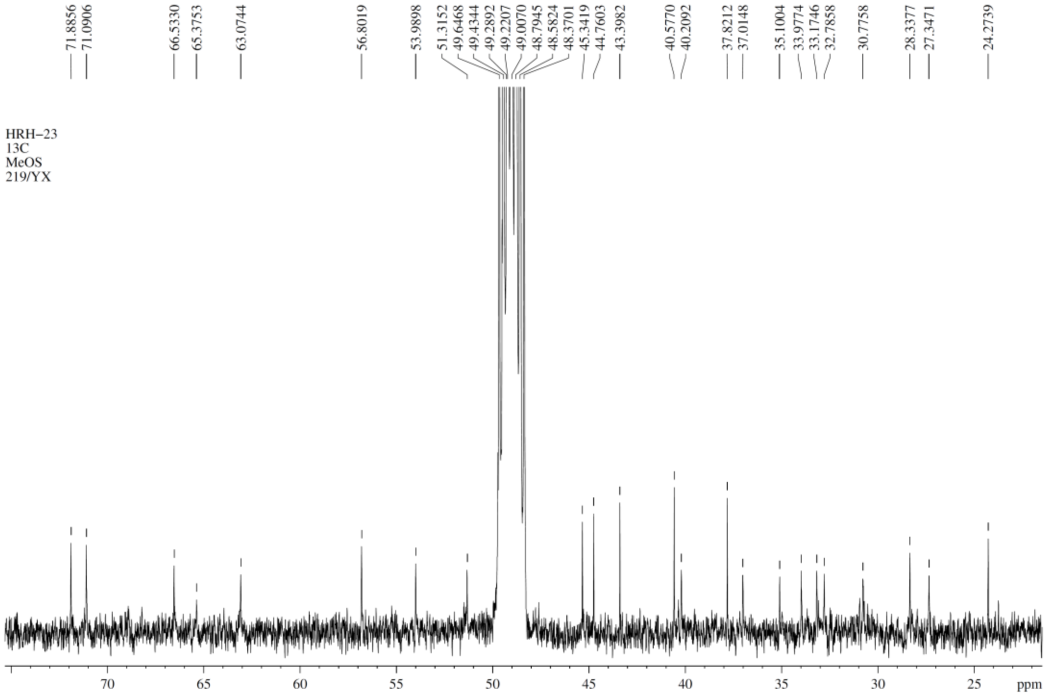

Figure S34: ^{13}C NMR extended spectrum of Compound **3**

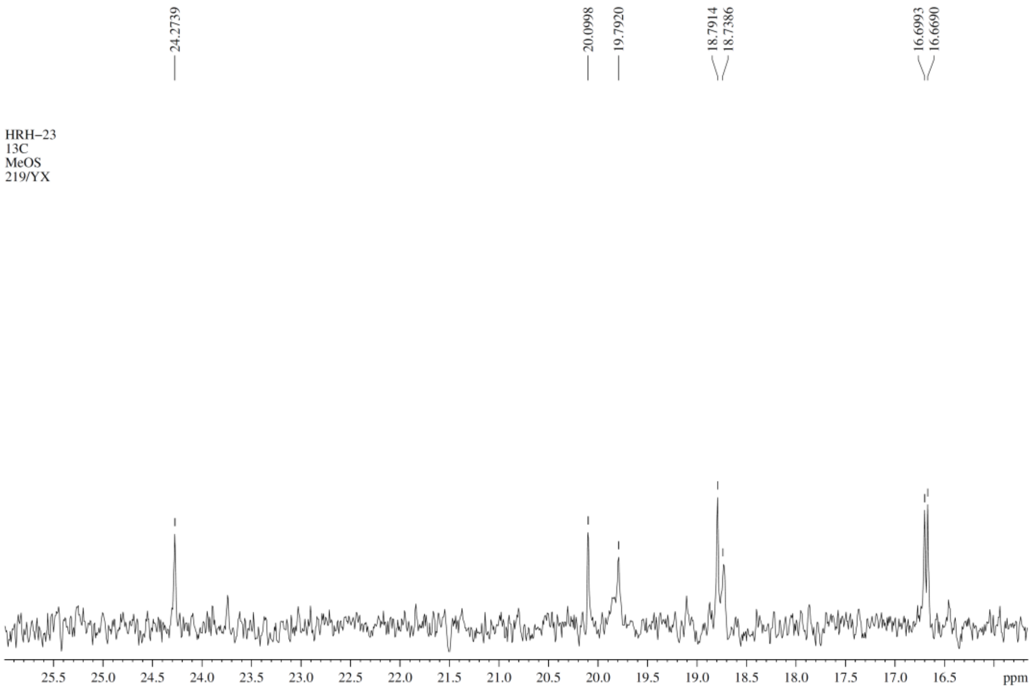

Figure S35: ^{13}C NMR extended spectrum of Compound **3**

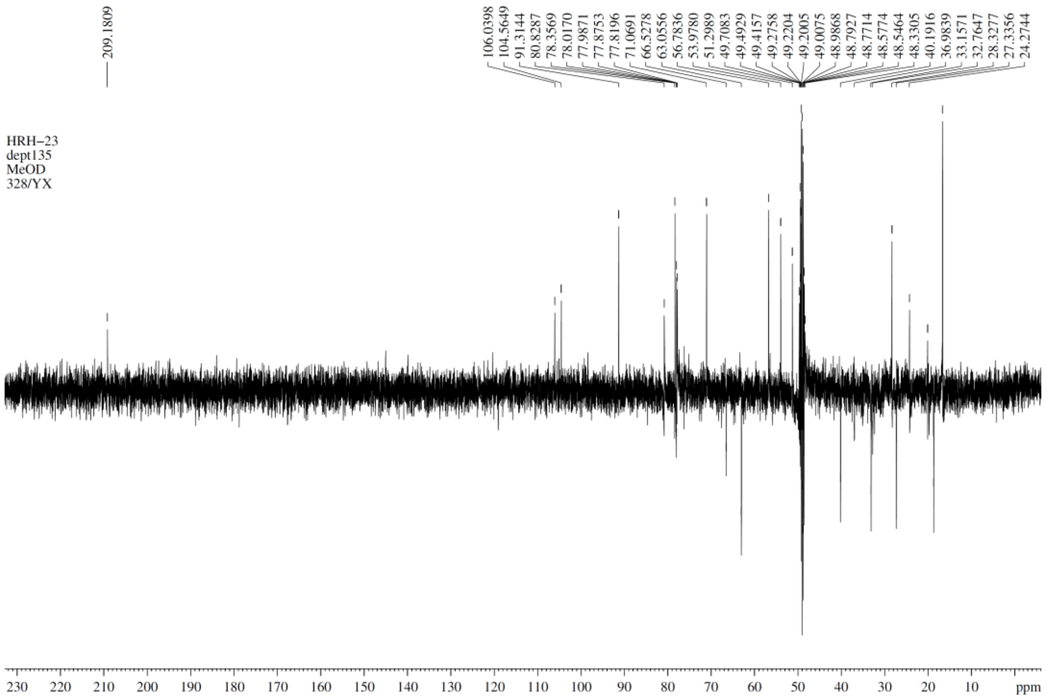

Figure S36: DEPT-135 spectrum of Compound **3**

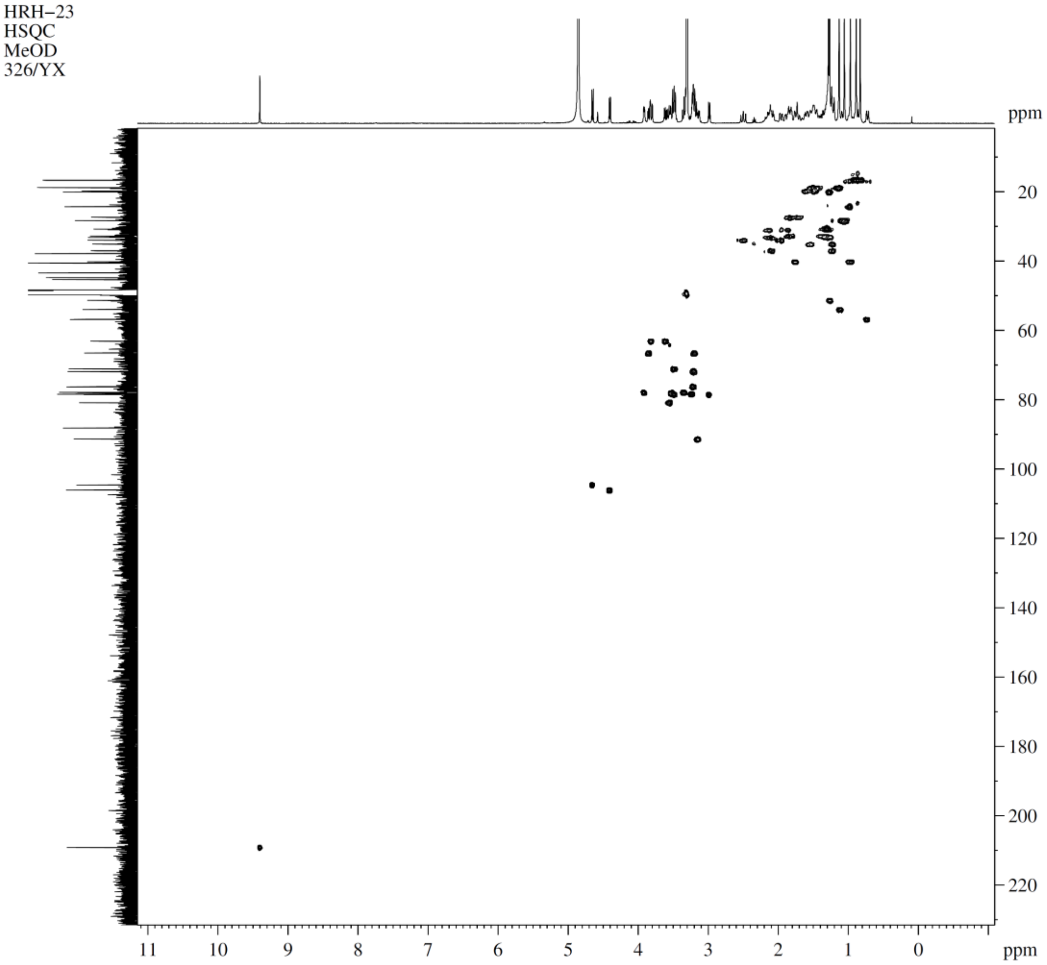

Figure S37: HSQC spectrum of Compound **3**

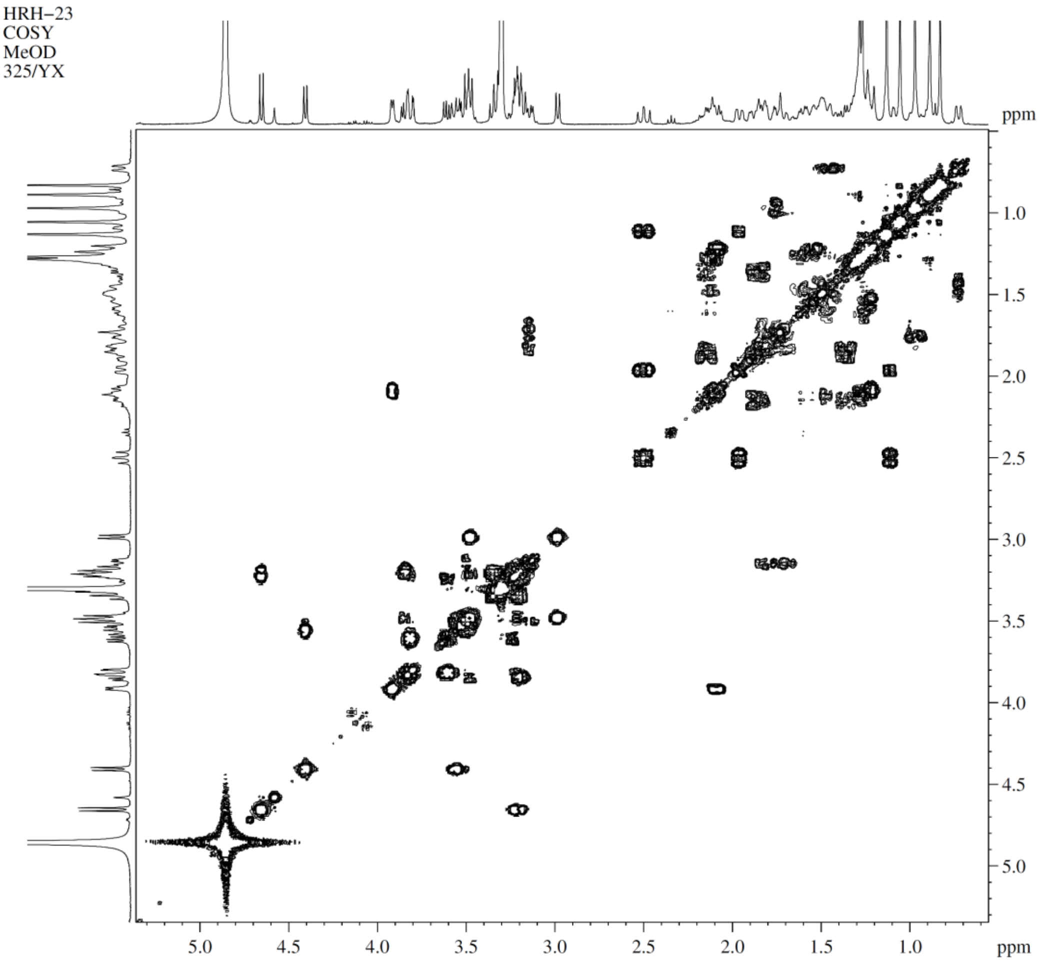

Figure S38: ^1H-^1H COSY spectrum of Compound **3**

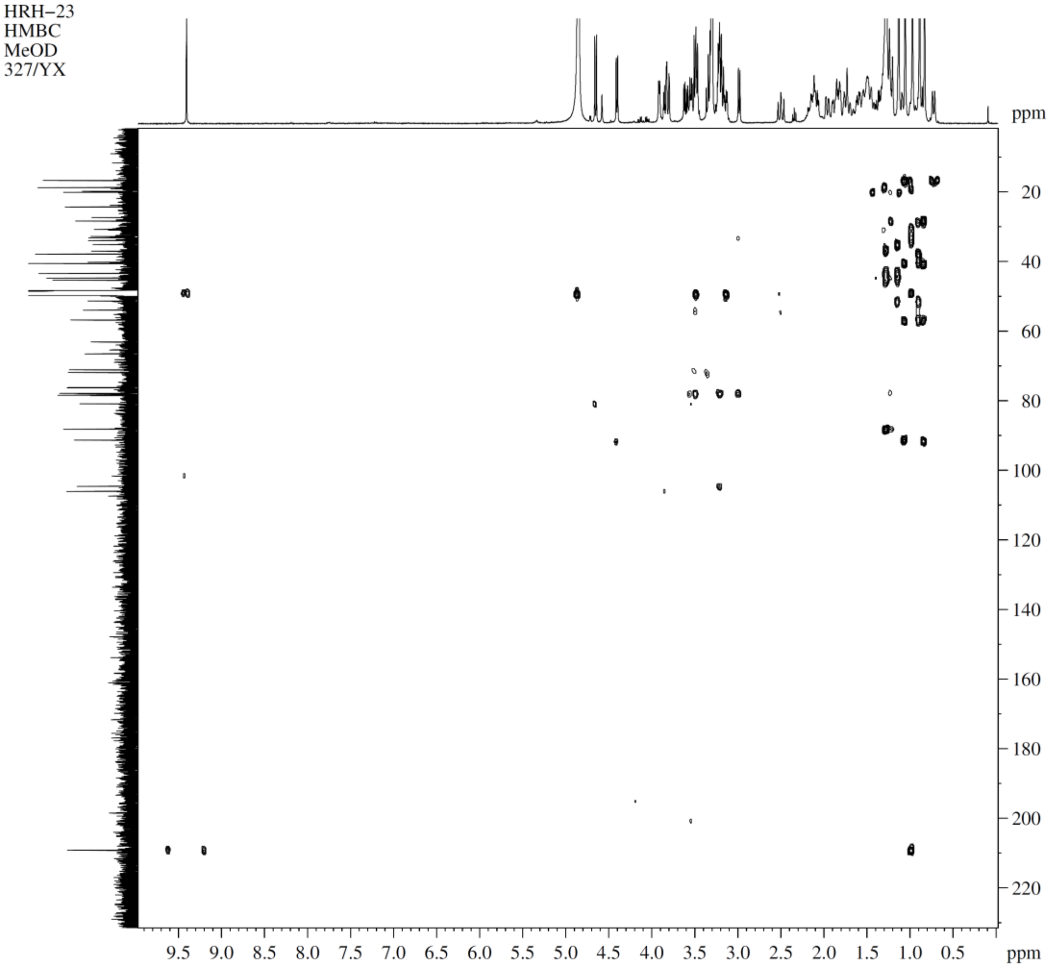

Figure S39: HMBC spectrum of Compound **3**

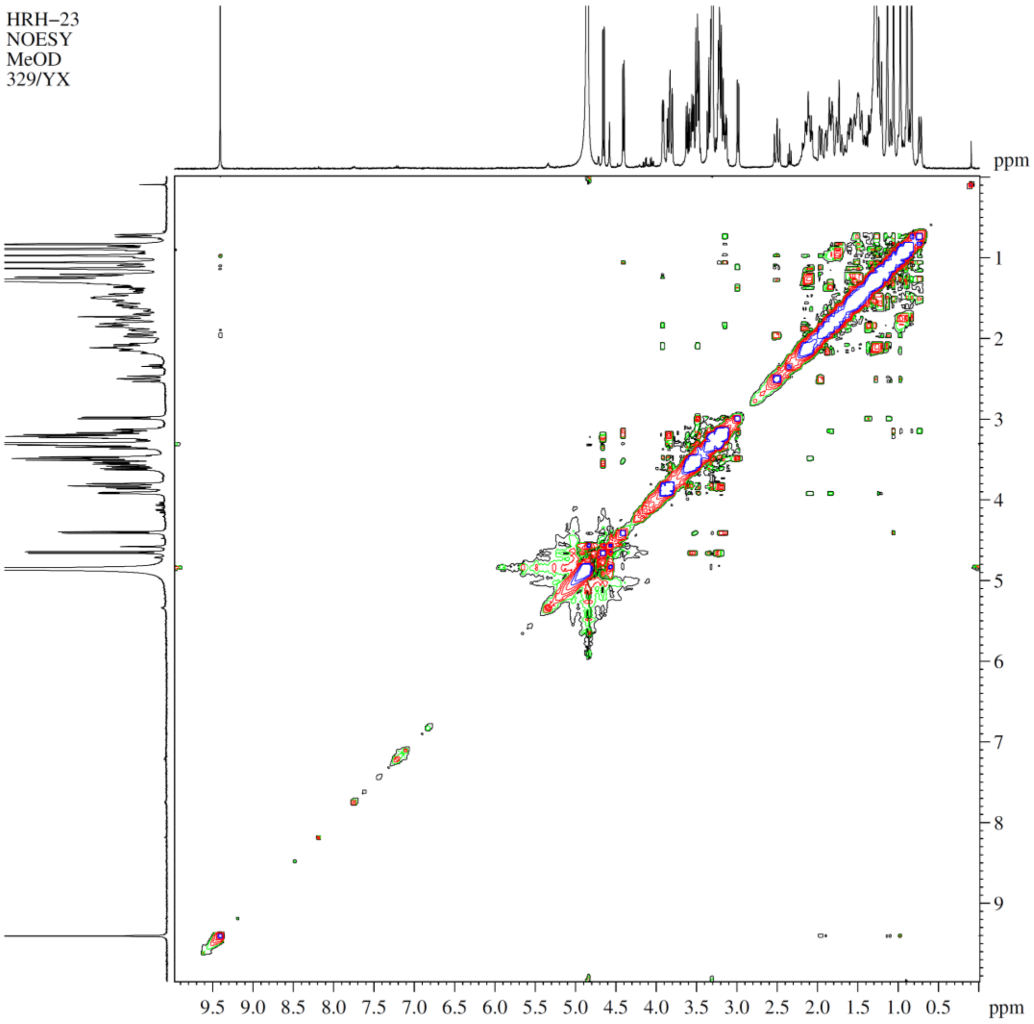

Figure S40: NOESY spectrum of Compound **3**

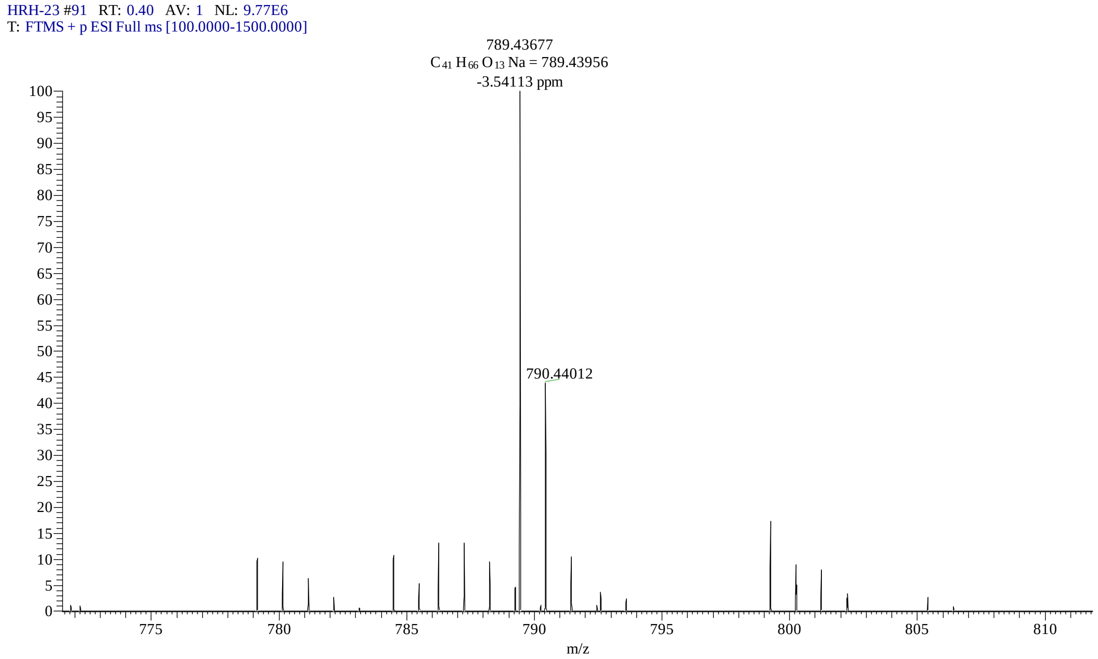

Figure S41: HR-ESI-MS spectrum of Compound **3**

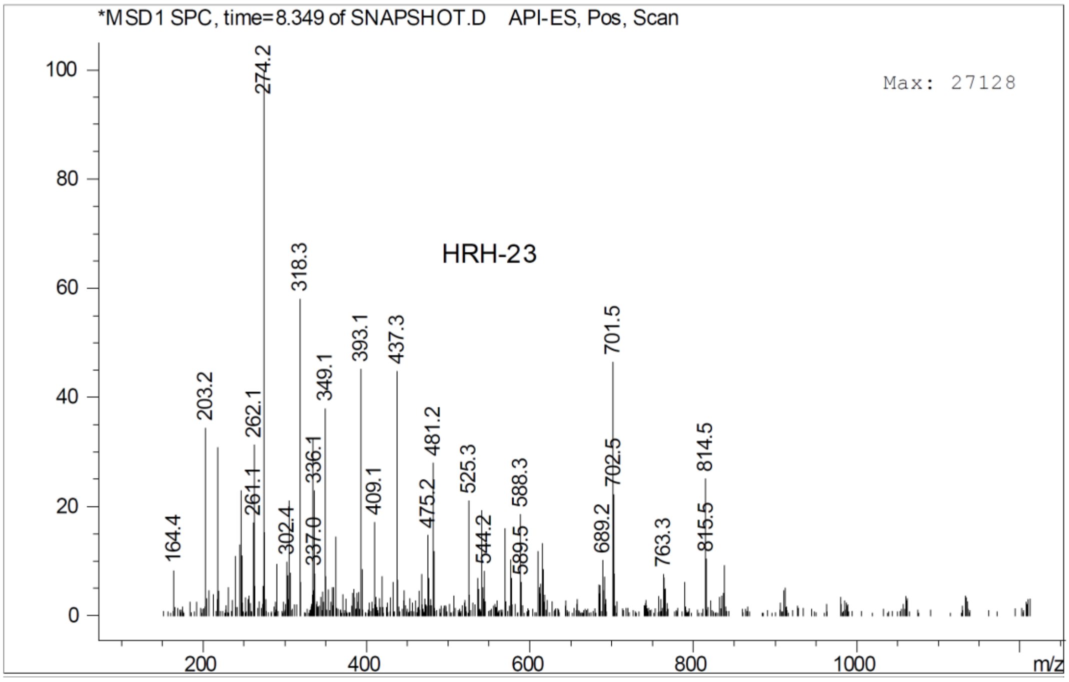

Figure S42: ESI-MS spectrum of Compound **3**

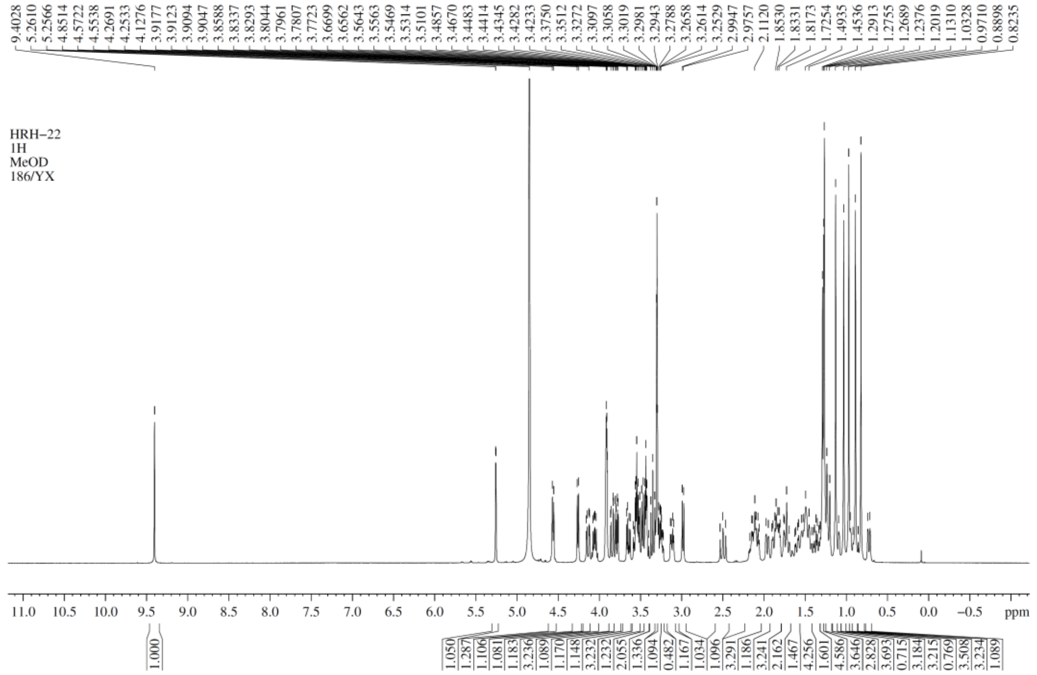

Figure S43: ^{1}H-NMR spectrum of Compound **4**

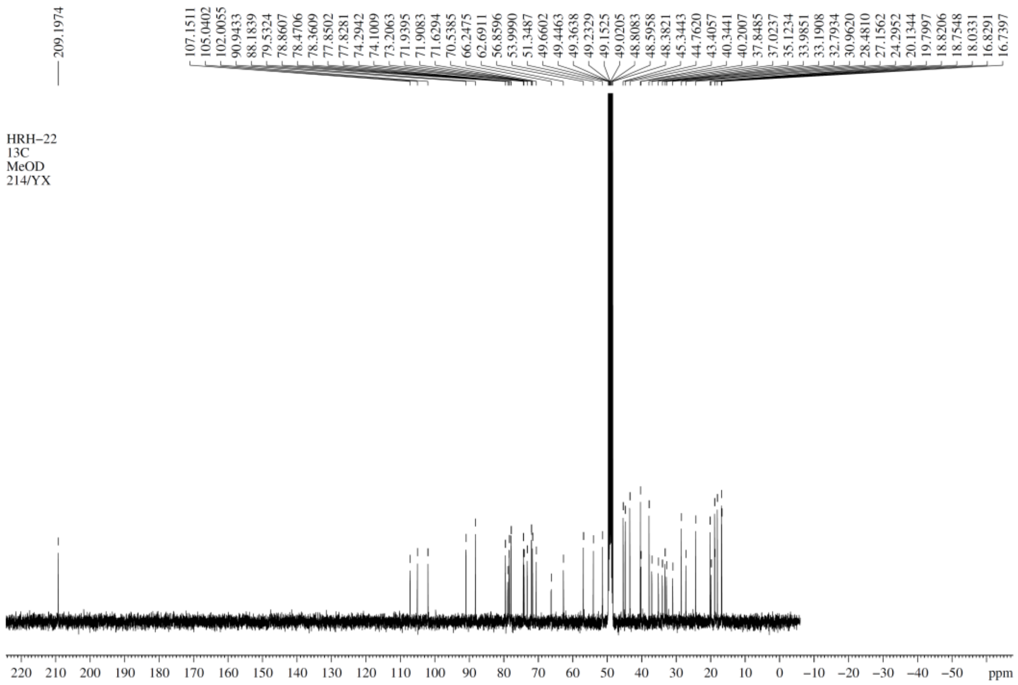

Figure S44: ^{13}C NMR spectrum of Compound **4**

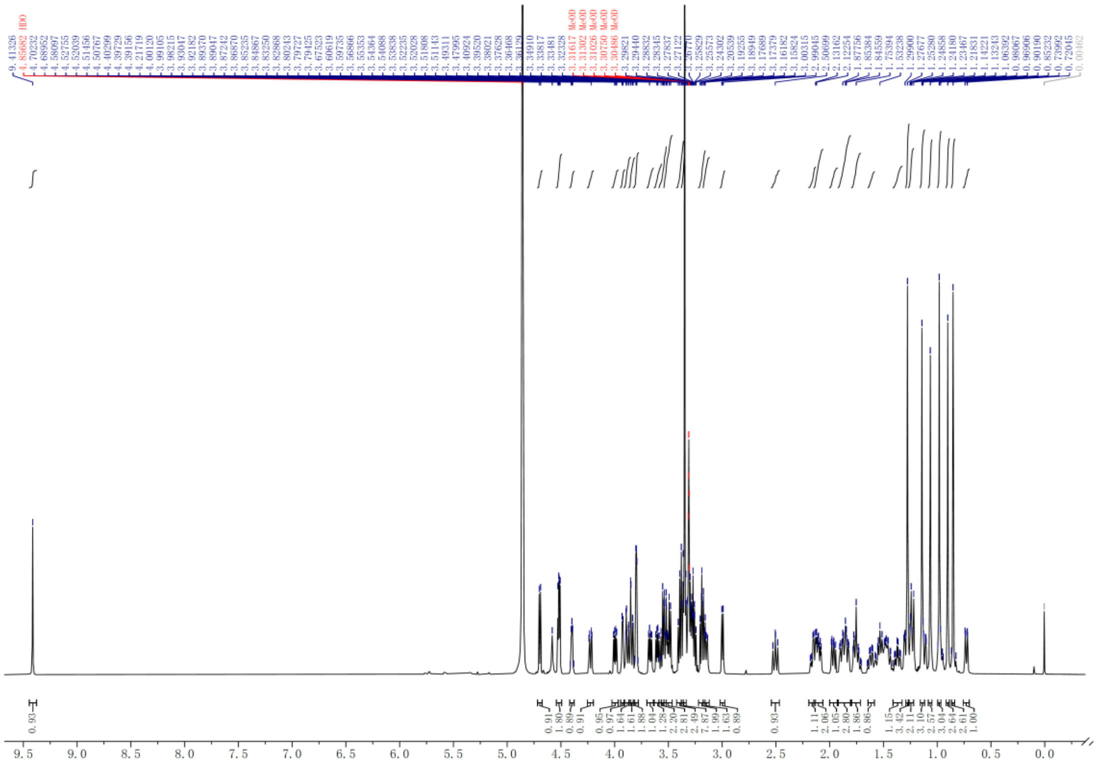

Figure S45: ^{1}H-NMR spectrum of Compound **5**

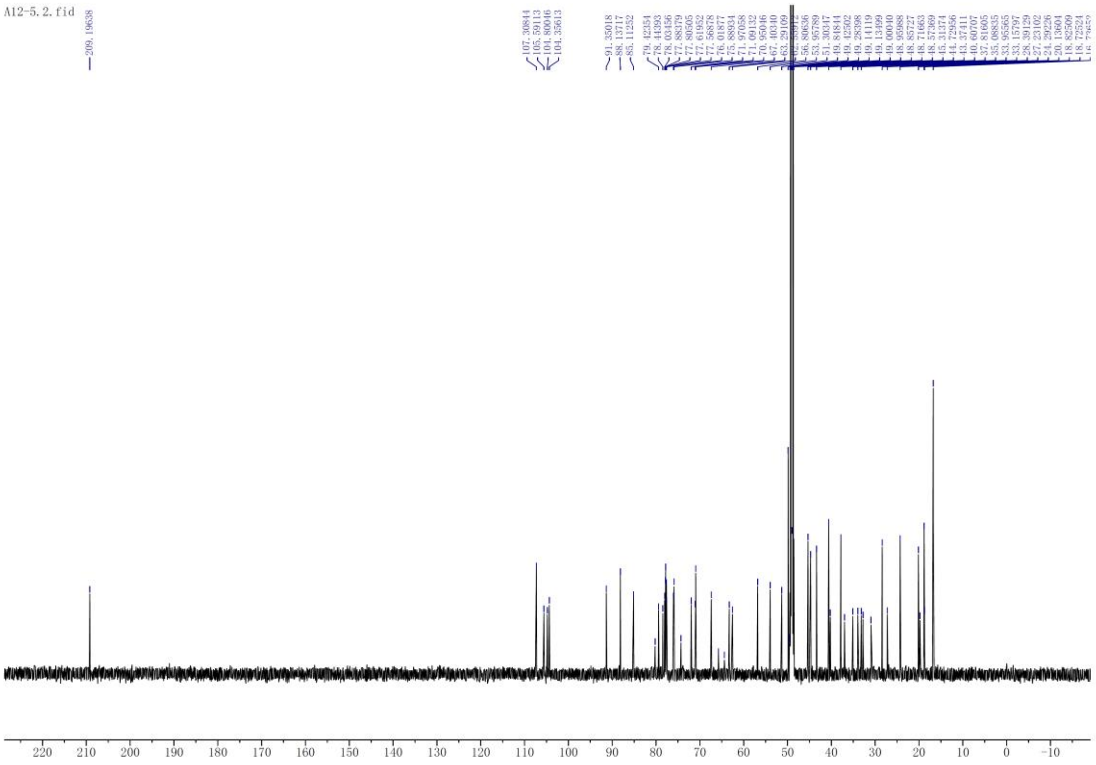

Figure S46: ^{13}C NMR spectrum of Compound **5**

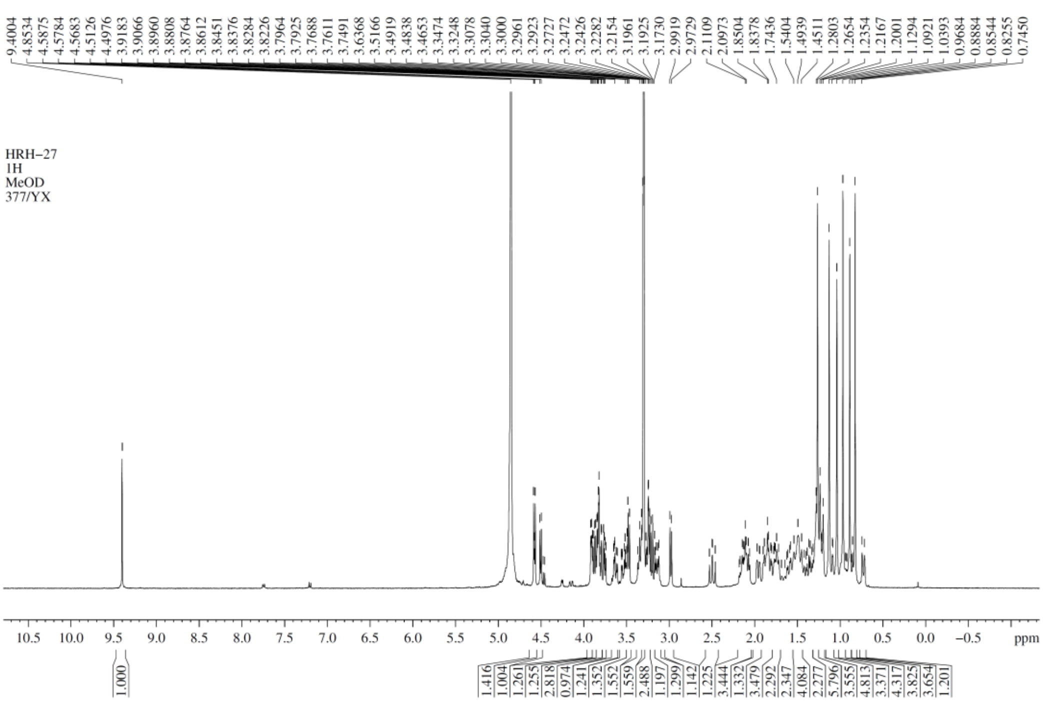

Figure S47: ^{1}H-NMR spectrum of Compound **6**

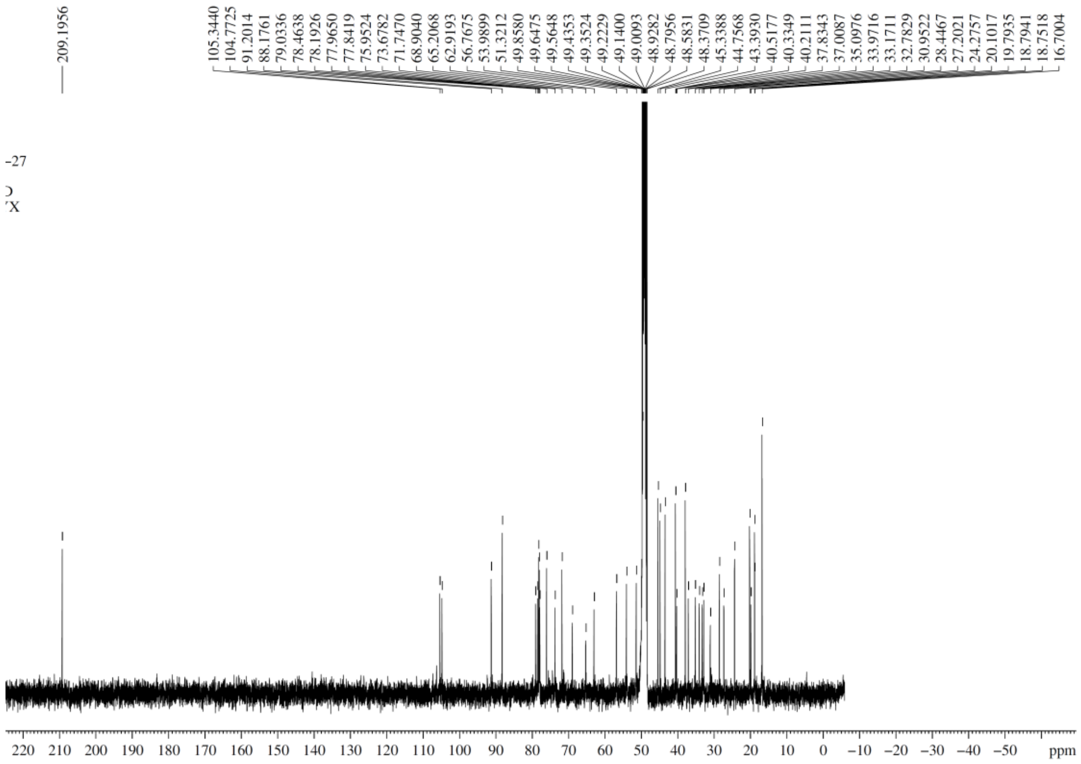

Figure S48: ^{13}C NMR spectrum of Compound **6**

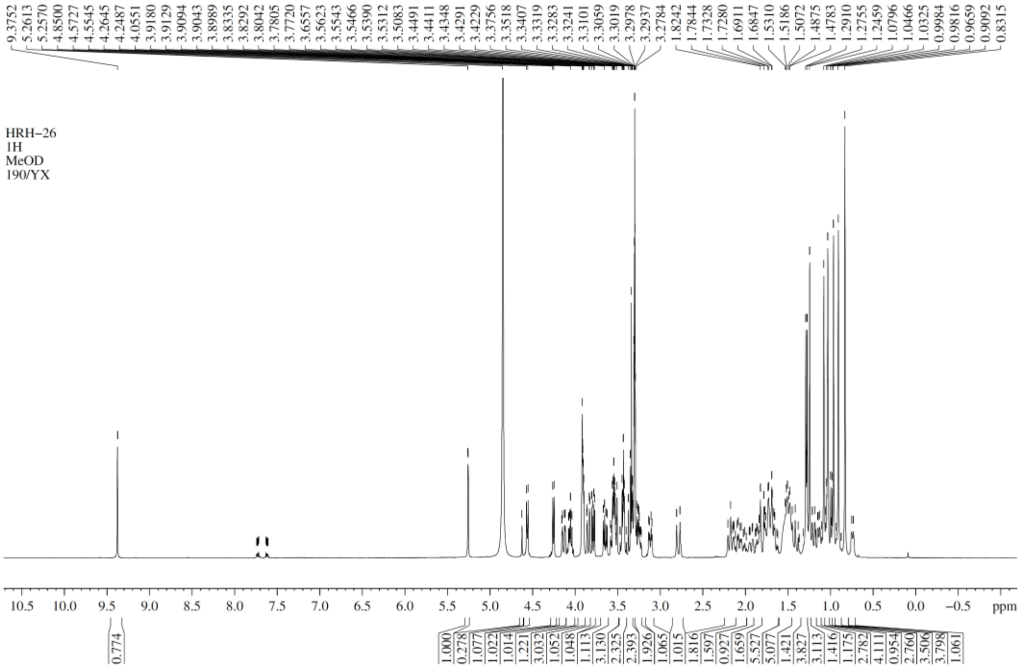

Figure S49: ^{1}H-NMR spectrum of Compound **7**

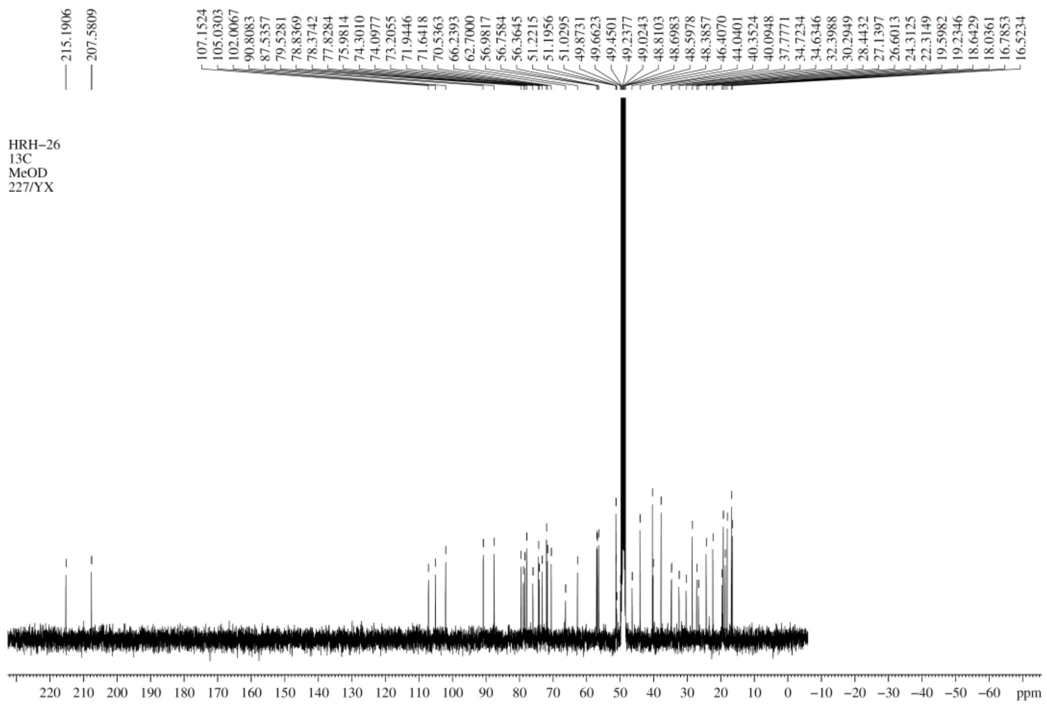

Figure S50: ^{13}C NMR spectrum of Compound **7**

Table S1 ^{13}C-NMR (100 MHz) Data of **4-7** in CD_3OD

Position	4	5	6	7
**1**	37.8	40.2	40.2	37.8
**2**	27.1	27.2	27.2	26.6
**3**	90.9	91.3	91.2	90.8
**4**	40.3	40.5	40.5	40.1
**5**	56.8	56.8	56.7	57.0
**6**	18.8	18.7	18.7	18.0
**7**	35.1	33.9	35.1	34.6
**8**	43.4	43.3	43.4	40.3
**9**	54	51.3	51.3	51.2
**10**	40.2	37.8	37.8	34.7
**11**	19.8	19.8	19.8	19.5
**12**	33.2	35.1	33.2	30.2
**13**	88.1	88.1	88.2	87.5
**14**	45.3	44.7	45.3	46.4
**15**	37.0	37.0	37.0	44.0
**16**	77.8	77.8	77.8	215.2
**17**	44.7	45.3	44.7	56.3
**18**	51.3	53.9	54.0	56.7
**19**	34	30.9	34.0	32.3
**20**	49	49.8	49.0	49.0
**21**	31	33.1	30.9	28.4
**22**	32.8	32.7	32.7	34.6
**23**	28.5	16.7	28.4	27.1
**24**	16.8	28.3	16.7	16.8
**25**	16.7	16.7	16.7	16.5
**26**	18	18.8	18.8	19.2
**27**	20.1	20.1	20.1	22.3
**28**	78.4	77.6	78.0	75.9
**29**	24.3	24.3	24.2	24.3
**30**	209.2	209.2	209.2	207.6
**Ara-1**	107.1	104.8	104.8	107.1
**Ara-2**	74.3	80.2	79	73.2
**Ara-3**	73.2	74.3	73.6	74.3
**Ara-4**	78.8	78.4	68.9	78.8
**Ara-5**	66.2	65.0	65.2	66.2
**Glc-1'**	105	104.3	105.3	105.0
**Glc-2'**	79.5	85.1	75.9	77.8
**Glc-3'**	78.3	77.8	78.4	79.5
**Glc-4'**	71.6	71.1	71.7	71.6
**Glc-5'**	77.8	79.4	78.2	78.3
**Glc-6'**	62.7	62.6	62.9	62.7
**Glc-1''**		105.7		
**Glc-2''**		76.0		
**Glc-3''**		78.0		
**Glc-4''**		72.0		
**Glc-5''**		77.9		
**Glc-6''**		63.2		
**Xyl-1**		107.3		
**Xyl-2**		75.8		
**Xyl-3**		77.5		
**Xyl-4**		70.9		
**Xyl-5**		67.4		
**Rha-1**	102			102.0
**Rha-2**	71.9			71.9
**Rha-3**	71.9			71.9

**Rha-4**	74.1	74.1
**Rha-5**	70.5	70.5
**Rha-6**	18.7	18.6
